# AFM methods for studying the morphology and micromechanical properties of the membrane of human buccal epithelium cell

**DOI:** 10.1038/s41598-023-33881-x

**Published:** 2023-07-05

**Authors:** N. A. Torkhov, V. A. Buchelnikova, A. A. Mosunov, I. V. Ivonin

**Affiliations:** 1 Sevastopol State University, Sevastopol, Russia; 2 Tomsk State University, Tomsk, Russia

**Keywords:** Biophysics, Cell biology

## Abstract

Using AFM methods in air under normal conditions in a wide range of local force effects (${F}_{const}$< 40 μN) the relief, functional micromechanical properties (elasticity coefficient $K$, Young’s modulus $E$, elastic ${\Delta h}_{dfrm}$ and plastic ${\Delta h}_{stiff}$ deformations) and adhesive properties (work $A$ of adhesive forces ${F}_{adh}={F}_{adh}(x;y)$) of the membranes of living adult cells of human buccal epithelium were studied in the presence of a protective layer < 100 nm of buffer solution that prevented the cells from drying. Almost all geometric and functional characteristics of the membrane in the local approximation at the micro- and nanolevels are affected by size effects and obey the laws of fractal geometry. The Brownian multifractal relief of the membrane is characterized by dimension ${D}_{f}$ < 2.56 and irregularities < 500 nm vertically and < 2 μm horizontally. Its response to elastic (≤ 6 *nN*), active (6–21 nN), or passive (> 21 nN) stimulation (${F}_{const}$) is a non-trivial selective process and exhibits a correspondingly elastic ($K=$ 67.4 N/m), active ($K=$ 80.2 N/m) and passive ($K=$ 84.5 N/m) responses. $K=K({F}_{const})$ and $E=E({F}_{const})$ depend on ${F}_{const}$. Having undergone slight plastic deformations ${\Delta h}_{stiff}$ < 300 nm, the membrane is capable of restoring its shape. We mapped ($E=E(x;y)$, ${D}_{f}$ = 2.56; ${\Delta h}_{dfrm}={\Delta h}_{dfrm}(x;y)$, ${D}_{f}$ = 2.68; ${\Delta h}_{stiff}={\Delta h}_{stiff}(x;y)$, ${D}_{f }$ = 2.42, $A=A\left(x;y\right)$ and ${F}_{adh}={F}_{adh}(x;y)$) indicating its complex cavernous structure.

## Introduction

The non-neoplastic epithelium organized in several layers and extending to the nasal cavity, mouth and oropharynx, conjunctiva, mucous-associated lymphoid tissue (MALT) (which is part of the autonomous mucosal immune system of a person) is increasingly considered as a cellular and tissue material for non-invasive diagnostics of the state of the human body^1^. The cytological method is one of the ways to study the epithelium cells of oral cavity which are the buccal epithelium of mucous membranes. This method is based on the high sensitivity of buccal epithelium cells to the state of human health, which indicates its advantage. Functional pathological changes in the buccal epithelium cells, in particular, its morphology, correlate well with indices of impaired homeostasis of the body, which makes it possible to quickly assess its state. These cells are highly informative regarding the influence of various physical, mechanical, chemical, environmental factors and drugs on human body^2^. This can serve as a source of important diagnostic and prognostic information about the state of health, stress effects, the influence of environmental factors and xenogeneic intoxication, and pharmacology. Changes in the cells of the buccal epithelium cells can also be considered as markers of the pre-tumor state of body^2,3^. In addition, good accessibility and non-traumatic reproduction of their collection relative to the oral mucosa, as well as the simplicity and low cost of sample preparation, makes them a convenient biological object for the intravital diagnosis of most socially significant diseases^4^. The surfaces of the oral mucosa play an important role in the sensory formation of touch, smell and taste, which is largely reflected in the psychoemotional state of a person^5^. As a result, studies of functional pathological changes in the morphology of the buccal epithelium cells and micromechanical properties under the influence of external factors may significantly expand the capabilities and quality of this non-invasive diagnostic method.

It is known that the cell membrane is largely responsible for the state of the cell and the quality of its excitation mechanisms. It is one of the important organelles that determines the shape of the cell, ensures its integrity, controls its interaction with external objects, regulates the metabolism and energy exchange between the cell and the environment. At the same time, the integrity of the membrane is determined not only by the integrity of its geometric shape, but also by the unity (relationship) of its various functional characteristics, in particular, the relationship of chemical^6^, physicochemical^7^, physiological^8,9^, as well as physical^10,11^ excitation mechanisms. The latter largely determine the mechanical properties of living cells. While the tribological and adhesive properties of human buccal epithelium cells are relatively well studied^11^, their other micromechanical properties, such as stiffness (elasticity, characterized by the Hooke’s coefficient $\left[K\right]$ = N/m ≡ Pa), resistance to elastic deformation (Young's modulus $\left[E\right]$ = N/cm^2^), elastic ${\Delta h}_{dfrm}$ and plastic ${\Delta h}_{stiff}$ deformations have not yet received sufficient physical investigation. This is the motivation for conducting research in this field. Recall that the coefficient of elasticity (stiffness) $K$ connects in the linear Hooke's law $F=K\times \Delta z$ the elastic force *F* with the elongation of the elastic body $\Delta z$, where $K=ES/{l}_{z}$. Here $E$ is the Young's modulus, which characterizes the ability of an elastic sample to resist tension or compression, $S$ is the area of its cross section of the sample, and ${l}_{z}$ is its initial length.

Probe microscopy methods can be considered as one of the most convenient tools for cell biology, providing the study of eukaryotic cells membranes with submicron and nanometer resolution^5,7,10–17^. Probe research methods, in particular, atomic force microscopy (AFM), with a fairly wide range of functional and spatial resolutions, allow not only qualitative but also quantitative levels of comprehensive study of morphological and physical characteristics (in particular, micro- and nanomechanical) of living biological objects under normal conditions (normal atmospheric pressure and temperature) in air^5^ and in liquid media^12^.

For example, the use of the AFM and scanning microwave RF-microscopy to study the morphology and electrical properties of human buccal epithelium cells has made it possible to reveal the effect of salivary proteins on the hydrophobic and hydrophilic properties of local areas of the outer cell membranes. It allowed developing the basics for the mechanisms of film formation of the mucous membrane in the human oral cavity^5,7^.

The AFM studies carried out by the authors in ref.^12^ in the liquid medium of human intestinal Caco-2 cells revealed the effect of homeostatic changes on local elasticity (Young's modulus, $E=$ 3.77–10.95 kPa) and the permeability of their membranes, estimated by the value of their layer resistance > 100 Ohm/cm^2^—barrier functions. Control of the plasticity of human bronchial epithelial cells (16HBE cells) in close modes of the AFM force microscopy in the range of small (elastic) deformations of 10–40 nm showed close values of Young's modulus, $E$*,* approx. 5 kPa^13^. The study of drug-induced reorganization of actin filaments and microtubule elements involved in the formation of cancer cells cytoskeleton in U138 glioma showed their greater deformability (Young's modulus, $E=$ 2.52 ± 0.89 kPa), than that for non-malignant cells (Young's modulus, $E=$ 3.01 ± 2.21 kPa)^3^.

The disorders in the structure and functioning of the cytoskeleton, which are targets for anticancer therapy, described in ref.^3^, lead to the death of cancer cells. The authors of ref.^18^ pointed out a strong dependence of the stiffness of the $S$*. aureus* cell membrane on the intensity of external influence (5 pN–10 nN). For instance, for a cantilever with the $K=$ 0.01 N/m rigidity, the membrane rigidity is $K=$ 0.0134 ± 0.0068 N/m, and for a cantilever with a higher rigidity, $K=$ 0.07 N/m, this value is $K=$ 0.2062 ± 0.0039 N/m. It should be noted that in both cases the stiffness of the cell membrane exceeded the stiffness of the silicon cantilever beam. All this points to the complex mechanical organization of the cell membrane, which manifests itself only with its active stimulation with large force effect. In this regard, the authors of^10^ showed that the mechanical action on the membranes of human erythrocytes and neutrophils with a force of up to 125 nN is accompanied by nonlinear effects of elastic deformation–a significant increase in the values of the Hooke’s coefficient *K* and Young’s modulus *E* with a decrease in the zone of uniform bending of the membrane. It should be noted that the authors of^10^ divided the process of elastic deformation of membranes into three stages characterized by three deformation zones.

Despite the obvious progress in this direction, the amount of experimental data on the response of the human buccal epithelium cell membranes to external mechanical stimuli, on the shape and geometry of their surface relief and its micromechanical properties, rather limited. In some cases, this is due to the use of insufficient resolution, in others—the lack of modern geometric methods and AFM techniques that allow recording a full range of Brownian and micromechanical surface parameters in a single measurement cycle. It is thus important to develop effective methods for quantitative control of how such cells perceive, transform and apply external mechanical signals^19–22^. Such properties of cells are the basis for atomic and molecular nanoengineering (manipulation of nanoobjects, creation of micro- and nanostructured devices) both on the surface and inside the cell.

The main goal is to develop and test effective methods for quantitative control of not only geometric (in particular, the relief), but also functional (for example, micromechanical) characteristics that are responsible for how the cell perceives, transforms, and applies external mechanical signals^18,23^. The study of such properties of cells is the basis for atomic and molecular nanoengineering (manipulation of nanoobjects, creation of micro- and nanostructured devices) both on the surface and inside the cell.

The aim of this work is to study the response of cell membrane to external mechanical stimuli using atomic force microscopy methods in wider range (up to 40 *µN*) of force effects (active and passive stimulation), surface morphology (in particular, the geometry of the relief $h(x;y )$) of the membranes of living cells of human buccal epithelium and mapping of its micromechanical properties (elasticity (Young's modulus, $E=E(x;y)$, elastic $\Delta {h}_{dfrm}= {\Delta h}_{dfrm}(x;y)$ and plastic $\Delta {h}_{stif}={\Delta h}_{stif}(x;y)$, deformations).

## Materials and methods

### Object of study

The object of the study was live freshly harvested buccal epithelium cells of the human oral cavity (hereinafter referred to as cells) obtained by liquid cytology. This method included the following steps: mechanical sampling (scraping) from the inner surface of the cheek of the oral mucosa by scraping with a sterile blunt spatula (a non-smoking donor was not recommended to consume hot and alcoholic beverages, food three hours before collecting cells, as well as any rinsing of the oral cavity), washing the scraping in a phosphate buffer (3.03 mM phosphate buffer with the addition of 2.89 mM calcium chloride with a volume of 5 ml, pH 7.0), placing this mixture in a test tube and separating its contents in a centrifuge for 5 min with an acceleration of 1700*g* (5000 min^−1^), selection of aliquots from a centrifuge tube of a buffer solution containing a suspension of living buccal epithelial cells. The cells remained viable as long as they were covered with a phosphate buffer. The phosphate buffer is isotonic in its parameters. Before placing the cells in it, the buffer was not subjected to additional filtration after cooking.

The epitaxial structure of p-type silicon was used as a substrate material p-p^+^- Si{111} with the size of the irregularities < 20 nm. To increase the hydrophilic properties and the adhesion of the surface the silicon structure was treated in vapors of hexamethyldisilazane.

**Figure 1:**
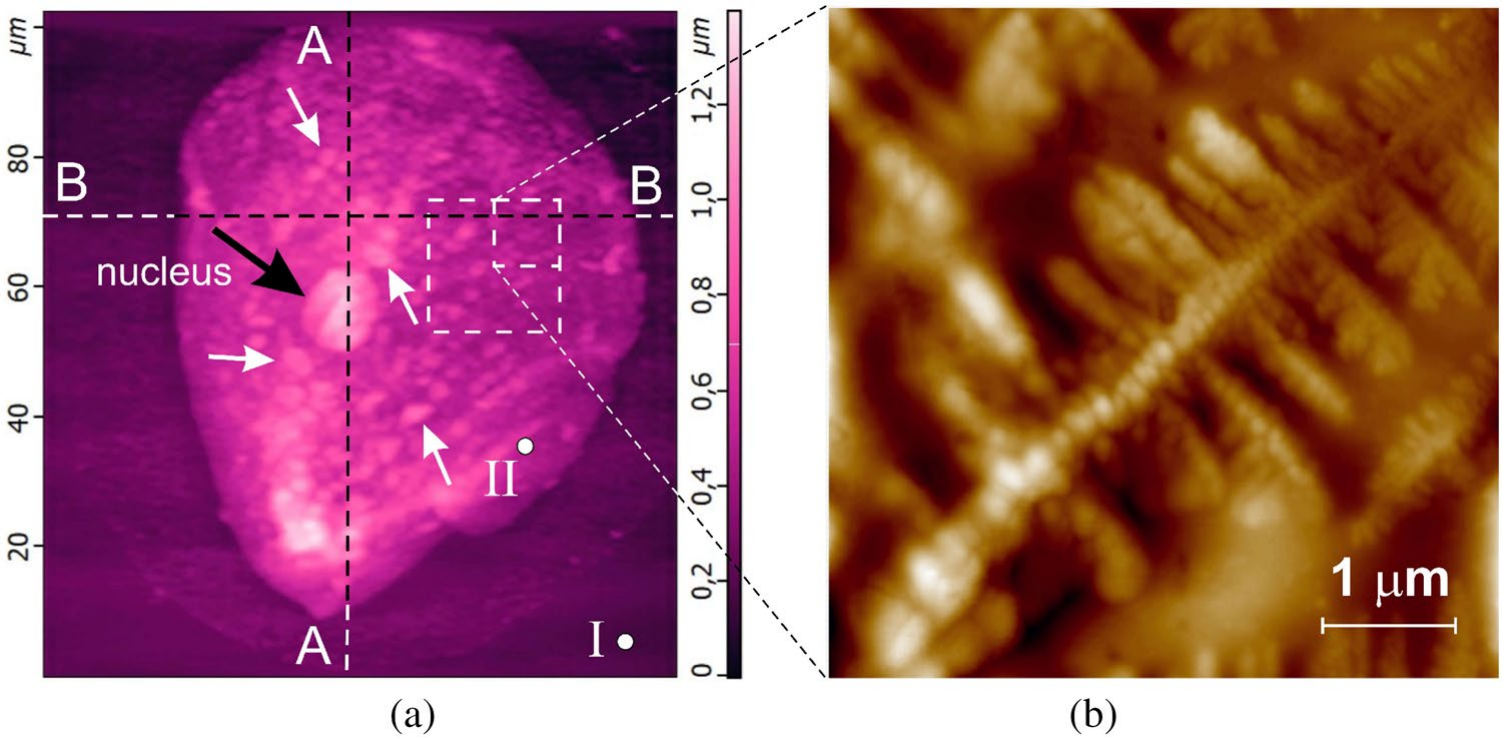
Raster images of 100 × 00 µm of an epitaxial Si{111} surface area with human buccal epithelium cells located on it, obtained by contact AFM scanning in constant force mode: relief $h(x;y)$ (the black arrow indicates the location of the nucleus, white arrows—some organelles and micronuclei inside the cell) (**a**), and a 5 × 5 µm relief of the surface area of the shell of immobilized cell of the buccal epithelium after 2.5 h and the destruction of the initial mosaic structure of the shell, coagulation of its protein and the formation of peptidoglycan dendrite (**b**).

After placing a drop of a cell aliquot on the epitaxial surface of silicon Si{111}, it was dried in air at normal atmospheric pressure and temperature $T$ ≤ 40 °C. The cells in the aliquot were naturally settled on the epitaxial silicon surface and remained on it in this form for 3–4 h after the evaporation of the main amount of moisture (Fig. 1a). This was indicated by irreversible changes in the state of the surface and morphology of their membranes that occurred, according to the studies, after 3–4 h of exposure of the cells to air, or longer-over 20 min of drying at $T$ ≤ 40 °C (Fig. 1b). The drying regime left an adsorption layer of a buffer solution on the cell surface, which maintained their viability during a relatively long exposure to air. Cell viability of the buccal epithelium cells was assessed by trypan blue staining (dye exclusion method). This dye does not penetrate the cell through an intact membrane, and therefore does not stain live cells. Most of the cells in the aliquot droplets on the epitaxial silicon surface retained their viability, i.e. were resistant to the dye, for 2.5 h. The state of cell viability was also controlled by their appearance (Fig. 1b).

### AFM methods

**Figure 2:**
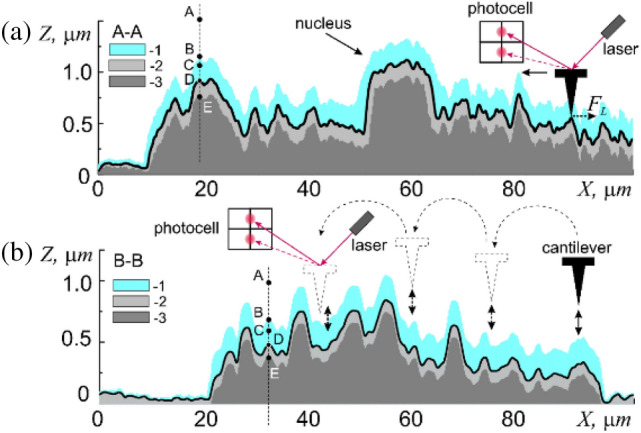
Cross-sectional profiles, A-A and B-B, shown in Fig. 1a of human buccal epithelium cell (1—adsorption layer, 2—elastic region and 3—the area of plastic deformation). Schematic representations of the AFM operation principles: contact (**a**) and hybrid (**b**) scanning modes. Due to the fact that the vertical and horizontal irregularities of the surface of the epithelial cell significantly exceed the thickness of the adsorption layer (~ 100 nm) in general, it will repeat the contour of the surface.

The studies of the surface morphology of membranes of human buccal epithelium cells and their micromechanical properties were carried out in atmospheric air under normal conditions using an NTEGRA-SPECTRA atomic force microscope (AFM) in contact (Fig. 2a, constant force mode ${F}_{z}={F}_{const}$) and hybrid (Fig. 2b, periodic exposure mode with a force ${F}_{z}={F}_{const}$) scanning modes in the Center for Collective Use “Molecular Structure of Matter” of Sevastopol State University. As a measuring probe, HA-FM/W_2_C cantilevers were used, which represent a micromechanical silicon device consisting of a rectangular 3.6 × 1.6 mm silicon base 0.4 mm in thickness in the center of which a beam was formed being 183 μм long, 34 μм wide and 3 μм thick. The upper surface of the beam was covered with a reflective gold coating.

From the lower side of the free end of the beam, a pyramidal 12 μм high and with the apex angle of $\alpha \approx$ 22° needle coated with a high-strength layer of tungsten carbide W_2_C was formed. The top of the pyramidal needle was a point with a radius of curvature $r=$ 5 nm (hereinafter referred to as the probe). The design parameters of the needle and the conditions for carrying out probe measurements (large deformations and the presence of adhesive forces) imposed certain restrictions on the choice of models for the contact interaction of the tip (probe) with the surface.

**Figure 3:**
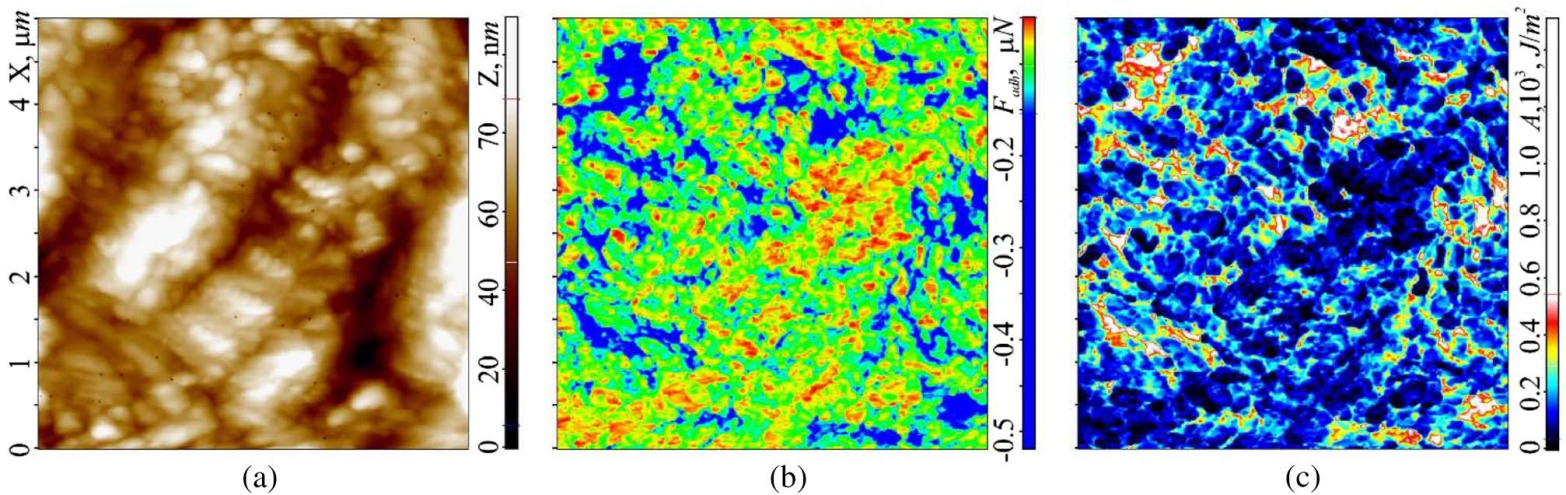
Bitmap (raster) AFM images of a 5 × 5 μm region of the membrane surface of a human buccal epithelium cell obtained in a hybrid scanning mode in the region of elastic deformations $\Delta z<$ 100 nm: (**a**) relief $h=h(x;y)$, (**b**) adhesive forces ${F}_{adh}={F}_{adh}(x;y)$ and (**c**) work of adhesive forces $A=A(x;y)$.

**Figure 4:**
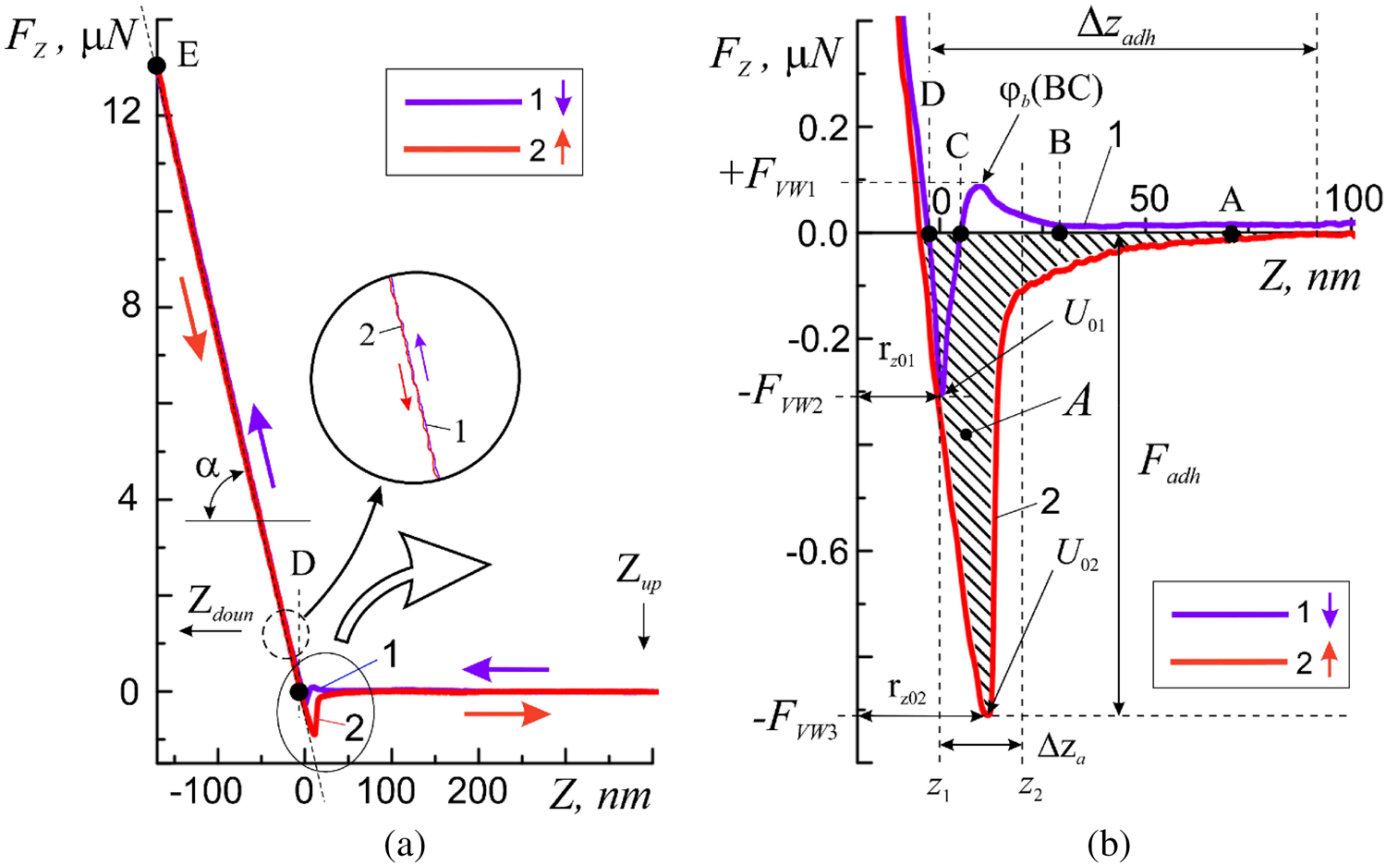
Calibration curves of attraction 1 and recoil 2 of the atomic force microscopy method (AFM) obtained for the epitaxial surface of a silicon substrate in the presence of an adsorption layer of a buffer solution (**a**) and a more detailed image of the same curves in the region of the adsorption layer (**b**).

In this case, the generally accepted Hertz model of the interaction of the probe with the surface for determining the micromechanical parameters of membranes was not suitable for a number of reasons. On the one hand, it was originally intended to describe the interaction force *F* of a solid probe with a semi-infinite elastic space in cases of small elastic deformations commensurate with or slightly exceeding *r* (usually $\Delta z$ < 30 nm). In our case, this contradicted the conditions for carrying out measurements under large force effects, under which $\Delta z$ > 30 nm $>r$ On the other hand, the Hertz model does not take into account the effect of adhesive forces on the interaction between the probe and the surface, which also contradicts the measurement conditions^11^. These requirements are best met by the so-called DMT-model (Derjagin-Muller-Toropov), used to calculate the values of micromechanical parameters ($K$ and $E$) under large force effects $F={F}_{const}$, taking into account adhesive forces in cases where the dimensions of the elastic deformation of the surface Δ*z* at the point *(x;y)* significantly exceed the radius of the needle tip $\Delta z\gg r$^24,25^:

1 $$F\left( {\Delta z} \right) = Er^{1/2} \Delta z^{3/2} + 2\pi Ar,\quad K\left( {\Delta z} \right) = Er^{1/2} \Delta z^{1/2} + \frac{r}{{a^{2} }}F_{adh} ,$$

where ${F}_{adh}$—adhesion force (Fig. 3b), ${A=F\Delta z}_{adh}$—work of adhesion forces (Fig. 3c, similarly to the surface tension $F= {F}_{adh}/2\pi {a}^{2}$ are traditionally measured in $\left[F\right]=N/{m}^{2}$ and in $\left[A\right]=J/{m}^{2}$), $a= \sqrt{r\Delta z}$ is the radius of the contact pad of the probe with the surface. The physical meaning of the work of adhesive forces is determined by the reversible thermodynamic work that must be expended to break the adhesive bond between the two bodies—reaching the distance ${\Delta z}_{adh}$ (Fig. 4b), divided by the area of their contact—the contact pad with a radius *a*. Young's modulus $E$ at the point $\Delta z$ can be determined from (1) by a graphical method.

As follows from the DMT model (1), the functional dependence $F=F(\Delta z)$ of the force of elastic interaction of a solid probe with the surface in the region of reversible elastic deformations DE (Fig. 4a, DE), in the general case, is no longer required to have a linear dependence, as, for example, in Hooke's law in which the reaction force is directly proportional to the change in the linear size $\Delta z$:

2 $${F}_{H}={K}_{H}\times \Delta z,$$

where $\left[{K}_{H}\right]$ = N/m is the elasticity coefficient, or Hooke's coefficient ($H$—Hooke's law).

At the same time, in the area of action of the adhesive forces AD (Fig. 4b), when $\Delta z<{\Delta z}_{adh}$, both the adhesive forces ${F}_{adh}={F}_{adh}(z)$, and the work $A=A(z)$ performed by them may depend nonlinearly on the coordinate "*z*". Unlike Hooke's law, in which the coefficient ${K}_{H}= Const$ is a constant value, the elasticity coefficient $K$ in the DMT model in the general case depends on the adhesive forces ${F}_{adh}={F}_{adh}(z)$ and has a functional dependence on the coordinate $K= K(z)$ (1 ). Based on this, in the DE region, the relation $F= C\int K(z)dz$ (*C* is some constant), that is, for each of the “*z*” coordinates, the coefficient $K(z)=dF(z)/dz$ is, as in Hooke's law, the coefficient of proportionality between the force and the coordinate but written in differential form.

In the general case, the adhesive forces on the membrane surface ${F}_{adh}={F}_{adh}(x;y)$ arise both due to the molecular electrostatic interaction between the probe and the surface of the elastic medium ${F}_{me}$, and due to the capillary interaction with a thin (units of nanometers) adsorption layer. In this case, the contribution of capillary forces ${F}_{cap}$→0 to the total probe—surface interaction can be neglected, since the probe does not leave the thick (~ 100 nm) adsorption layer during scanning in our experiments, which covers the entire membrane surface. In this case, any changes to the $|{F}_{adh}|$ will be associated with changes only to the first component of ${F}_{me}$. At the same time, any movement or exchange of particles in the system in which the probe interacts with the surface leads to an increase in its entropy, and the process itself becomes non-conservative. This means that in order to find the work of adhesive forces $A=A(x;y)$, it is necessary to know the probe trajectory, which is relatively easy to implement using AFM methods (Fig. 3c). Thus, the work of adhesive forces $A$ will be equal to the area of the figure bounded from below by the curve $F= F(x;y)$, and from above by the “$Z$” axis (Fig. 4b, A).

**Figure 5:**
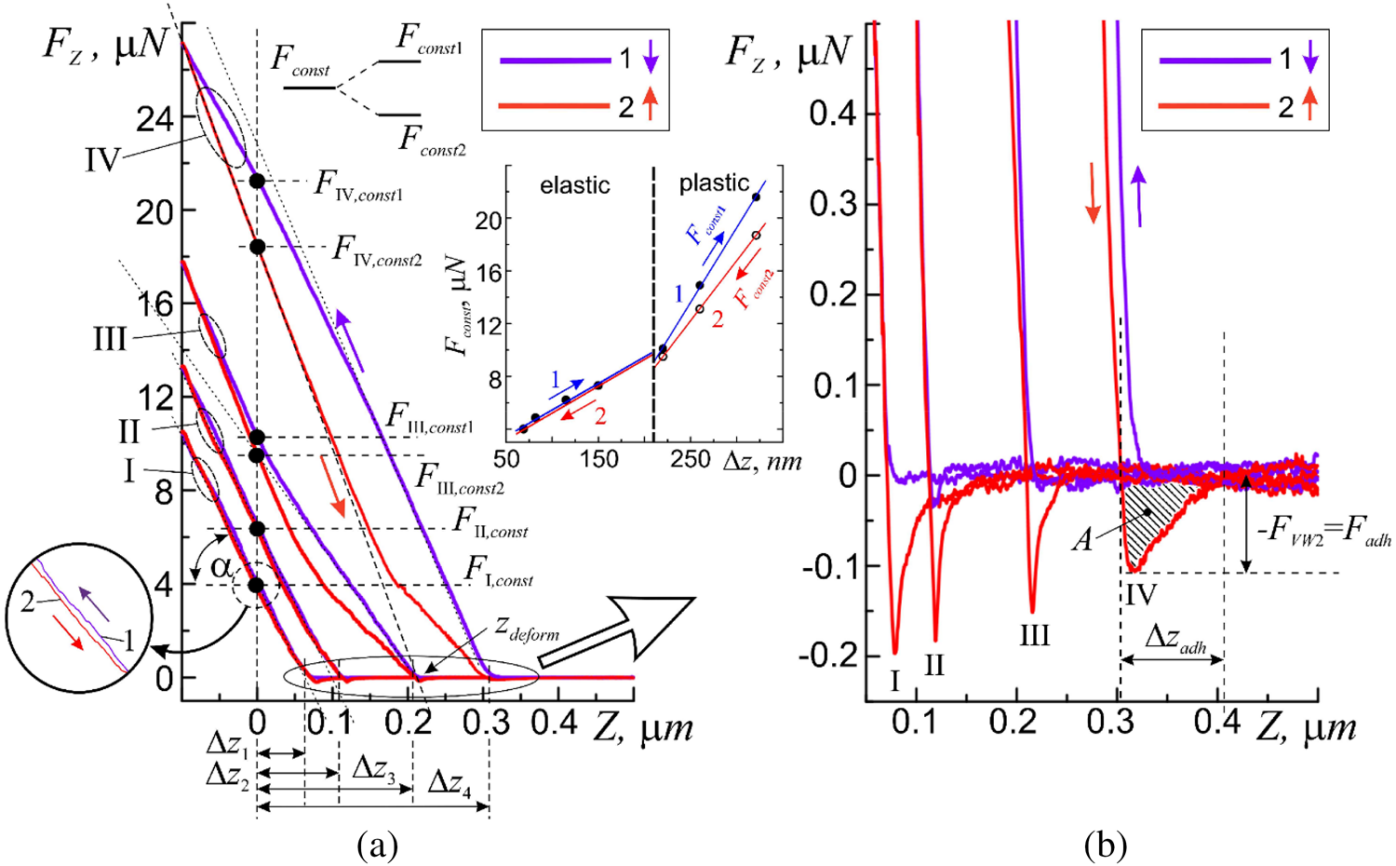
The approach 1 and withdrawal 2 curves of the AFM force spectroscopy method obtained on the membrane surface of human buccal epithelium cell at one point for the following values of the initial force effect: I—${F}_{\mathrm{I},const1}$ = 4 µN, II—${F}_{\mathrm{II},const1}$ = 6.2 µN, III—${F}_{\mathrm{III},const1}$ = 10.1 µN and IV—${F}_{\mathrm{IV},const1}$ = 21.6 µN (**a**), and a more detailed image of the same curves in the region of the adsorption layer (**b**). Inset—functional dependencies ${F}_{const}={F}_{const}(\Delta z)$: dots—experiment, lines—approximation, 1—at attraction, 2—at recoil, elastic—region of elastic deformations, plastic—region of plastic deformations.

Quantitative estimates of the process of local interaction of the probe with the surface were carried out by force spectroscopy by taking the force curves of attraction ${F}_{z}={F}_{z}(Z\downarrow )$ and recoil ${F}_{z}={F}_{z}(Z\uparrow )$ in the contact scanning mode (Figs. 4 and 5). The acquisition time for each 993-point attraction/recoil curve was one second, which was sufficient for the relaxation of the probe-surface mechanical system. These curves describe the functional dependence of the pressing force ${F}_{z}$ on the deformation value $\Delta z$ of the surface at the point (*x;y*). Constant values of ${F}_{const}$ were provided by the feedback between the value of the scanner piezotube extension along the “$Z$” axis and the photodiode matrix in the AFM control system. In this scanning mode, a constant mechanical contact of the probe with the surface is maintained at a constant (static) pressing force ${F}_{const}$ (Fig. 2a). An important option of this mode is the ability to directly control the value of ${F}_{const}$ in static mode.

In order to simultaneously study the geometric and functional characteristics of the cell membrane surface, we used the hybrid scanning mode, which currently belongs to one of the most advanced probe methods for studying surfaces, because it combines the advantages of both contact and semi-contact methods (Fig. 2b). This combination allows, in a single measurement process, together with the surface relief $h=h(x;y)$, to determine and map the set of its functional characteristics, including mechanical characteristics (elasticity coefficient $K=K(x;y)$*,* Young's modulus $E=E(x;y)$, elastic ${\Delta h}_{dfrm}={\Delta h}_{dfrm}(x;y)$ and plastic ${\Delta h}_{stiff}={\Delta h}_{stiff}(x;y)$ deformations) and adhesive (work $A=A(x;y)$ of adhesive forces ${F}_{adh}={F}_{adh}(x;y)$) characteristics. Briefly, the essence of this method lies in the fact that during scanning, with the step-by-step movement of the cantilever at each point, after establishing the specified force ${F}_{const}={F}_{z}(z=0)$, the curves of attraction ${F}_{z}={F}_{z}(z\downarrow )$ and recoil ${F}_{z}={F}_{z}(z\uparrow )$ in a given interval along the “$Z$” axis (Fig. 2b) are obtained. The values of functional characteristics are extracted by mathematical processing of force curves in the vicinity of the point *z*_0_.

The scanning time of one region with a resolution of 250 × 250 points was 7 min and 10 s. The relative humidity of the atmosphere was controlled by precision sensors and maintained at a constant level of ~ 35% during the scanning process, which ensured sufficient reproducibility of the scanning processes. The effect of relative humidity, solution composition, and sample preparation methods on the results of AFM measurements require further studies^26^.

Immediately before carrying out quantitative measurements of the surface parameters, the cantilever beam elasticity coefficient was calibrated to *K*_*c*_ ≈ 5.6 *N*/*m* at its resonant frequency $f=$ 107 kHz, taking into account the spectrum of thermal vibrations^27,28^. The ${K}_{c}$ values were determined automatically from the frequency characteristics of the cantilever beam oscillations in the frequency range from 50 to 200 kHz. The cantilever was attracted to the surface in the “touch” mode, which provided a fairly accurate step-by-step approach of the probe to the surface. The AFM software, taking into account the calibration of the cantilever, makes it possible to extract as the reaction force of the support ${F}_{s}$ (s—support) not the relative values of the photodiode current, but its absolute values on the action of an external force from the probe ${F}_{s}=-{F}_{const}$. Therefore, the values of the coefficient of elasticity $K$ and Young's modulus $E$ found from the attraction/recoil curves describe precisely the micromechanical properties of the surface at the probe-surface contact point, and not the mechanical properties of the entire cantilever-surface system.

To analyze the lateral (in the (*x;y*) plane) irregularities of the relief and the functional characteristics, digital copies of the studied sections of the membrane surface were used. The digital copy of the surface relief was an array of numbers (*x;y;z*), which was obtained as a result of direct measurement of the coordinate along the “Z” axis at each point (*x;y*) of the studied area of the membrane surface. To obtain digital copies of functional micromechanical characteristics at each point (*x;y*) of the studied area, the measured values of these characteristics were plotted along the “Z” axis, respectively. At the same time, at each point (*x;y*), the values along the “$Z$” axis were found as an average of 5 measurements. This automatic function was provided by the AFM suit, which is equivalent to averaging a digital copy over 5 copies. Thus, the functionality of the AFM made it possible to set, with a predetermined accuracy, the geometric dimensions of the relief irregularities and the lateral distribution of the values of its functional characteristics. In this case, each measured coordinate point (*x; y; z*) on the surface of the studied area of the cell membrane had an unambiguous set of values for “$x$”, “$y$”, and “$z$”, which did not depend on the method of measurement and the method of mathematical processing, which made it possible to adequately interpret the results. In this way, three-dimensional digital copies of the studied surface area were obtained, which were subsequently studied by the methods of fractal geometry. Repeated scans of the studied areas completely verified (confirmed) the previous results.

The choice of the pressing force ${F}_{const}$ for mapping the mechanical parameters of the membrane in the contact and hybrid scanning modes was carried out after analyzing the spectral characteristics ${F}_{z}={F}_{z}(Z)$ obtained in the range of force actions from 100 nN to 21.3 μN.

Preliminary studies using the Kelvin Force Probe Microscopy showed that the halo around the cell presented in Fig. 1a is due to the difference in electrostatic potentials Δφ of the cell and the silicon surface and is not associated with a violation of the integrity of the membrane. The electrostatic field surrounding the cell, caused by the difference in electrostatic potentials $\Delta \varphi$, stimulates preferential precipitation from the buffer solution in the area of the halo during sample preparation when the aliquot is dried. The study of the electrical characteristics of the cell membrane requires more detailed independent studies and is not considered in this paper.

### Fractal surface analysis method

The analysis of the geometric properties of the relief of the cell surface membrane within the framework of the geometry of fractional dimensions by means of the mathematical methods of fractal geometry was carried out. In most cases, to determine the measures $M$ of fractal objects, only non-negative functions defined on families of sets of Minkowski spaces are considered. For example, a line (1D object) uses line segments, an area (2D object) uses squares, and a volume (3D object) uses cubes. This approach allows one to study the features of the geometry of an object, but does not allow one to establish a connection between this geometry and the functional properties of this object, which also depend on the size of the measuring scale. In the general case, the transition in mathematical formalism from the description of the geometric properties of fractal objects to the description of their functional characteristics is not trivial.

To study the relationship between the geometry of a Brownian (random) relief of a cell membrane and its functional characteristics, it is necessary to use the concepts of measure, metric, metric and normalized functional (linear) spaces, which allow, in the general case, to consider scalar and vector quantities, continuous functions and numerical sequences from a unified standpoint. At the same time, for the convenience of using functional spaces, geometric concepts and definitions are used. For example, to determine the measure $M$, it is possible to use different additive functions if they are defined on some normed linear space (hereinafter referred to as the functional space), the set of which at the same time satisfies all the axioms of the linear space: additivity, triangle, and zero distance. For such spaces, the concepts of continuity and norm are applicable—an analog of the distance between points in Euclidean space. For example, the distance between the functions ${\mathcal{F}}_{1}$ and ${\mathcal{F}}_{2}$ from the functional space is understood as the norm $\Vert {\mathcal{F}}_{1}-{\mathcal{F}}_{2}\Vert$. The elements of the functional space can be arbitrary mathematical objects: scalar numbers, vectors, matrices, singular self-similar (fractal) functions and self-similar (fractal) sets, which can be used to describe not only geometric, but also functional properties of objects^29,30^. For example, the surface of a cell membrane can be characterized not only by its relief shape and area, but also by the surface energy density, the concentration of an adsorbate–an external impurity, the electric charge density, and so on, which can be described not only by positive, but also by negative functions.

The investigated functional characteristics of the micromechanical parameters of cell membranes, such as Hooke's coefficient $K=F/\Delta z$ and Young's modulus $E=K{l}_{z}/S$ (where $S$ is the cross-sectional area of the elastic sample, and ${l}_{z}$ is its length) are determined on the functional space that is under study and satisfy the basic axioms of the linear space. It was shown in^29,30^ that the well-known mathematical apparatus of fractal geometry can also be applied to such functions.

The task of defining the measure $M$ of a functional characteristic of a biological object is to determine how many times $N$ the measured object embedded in a limited space ${R}^{D}$ can be filled with a certain measuring (calibration) object described by the function $l(\delta )=\gamma ({D}_{T}){\delta }^{{D}_{M}}$, where $\gamma$ is the normalization factor, $\delta$ is the dimensionless scale, ${D}_{M}$ is the Minkowski dimension (dimension of a bounded set in metric space). Then, according to ref.^31^, $N$ = $1/{\delta }^{{D}_{M}}$. Instead of ${D}_{M}$ we will operate with the concept of Hausdorff–Besicovitch dimension, ${D}_{H}$ (hereafter referred to as the Hausdorff dimension), which is close to the concept of Minkowski dimension, ${D}_{H}\approx {D}_{M}$. Thus, the dimension ${D}_{H}$ of a bounded set in metric space can be represented as

3 $${D}_{H}={D}_{M}=\underset{l\to 0}{\mathrm{lim}}\frac{\mathrm{ln}(\eta )}{\mathrm{ln}(\zeta )},$$

where $\eta$ is the minimum number of $N$ sets of $l$ linear size with which it is possible to cover (fill) the measured set when the diameter $l$ decreases by a factor of $\zeta$^31^.

Thus, the measure of a functional characteristic of a biological object $M$ can be written as:

4 $$M=N\left(\delta \right)l\left(\delta \right)=\gamma \left(D\right){\delta }^{{D}_{T}-{D}_{H}},$$

where $\delta =1/\zeta$, ${D}_{T}$ = 1, 2, 3 is a whole topological dimension.

In the case of self-similar (for example, fractal) sets, the Hausdorff dimension can be considered as the similarity dimension ${D}_{H}={D}_{S}$ or the fractal dimension ${D}_{f}$.

From (3), taking the logarithm of the right and left sides, was obtained the expression for the dimension ${D}_{f}$ expressed in terms of the measure, $M$, of the object:

5 $$D_{f} = \frac{{\ln \left[ {\frac{{M_{0} }}{{M_{i} }}} \right]}}{{\ln \left[ {\frac{{l_{i} }}{{l_{0} }}} \right]}} + D_{T} \,\,{\text{and}}\,\,M_{i} = M_{0} \left[ {\frac{{l_{i} }}{{l_{0} }}} \right]^{{D_{T} - D_{f} }} ,$$

where $m$ is the number of the similarity level.

An ideal fractal object is formed by nested disjoint sets of self-similar figures. The object can be characterized by the following parameters: local approximation limit $L$, fractal (fractional) dimension ${D}_{f}$, scaling coefficients $\zeta$ and $\eta$, the law of affine transformation ${x}_{i}=\mathrm{A}{x}_{i-1}$^31^. ${D}_{f}= {D}_{T}$ at $l\ge L$.

Most Brownian or chaotic objects in nature, which often include biological systems, are not self-similar in the literal sense of the word. We can only talk about certain statistical similarity and statistical self-affinity, i.e. the similarity in a certain interval of measuring scales, characterized by the average value of the fractal (fractional) dimension over the set. The investigated object can be characterized not by one, but by several, depending on the measuring scale, values of fractal dimension ${D}_{f}= {D}_{f}(l)$, i.e., the so-called multifractal objects^31^.

To determine the scaling coefficients $\zeta$ and $\eta$ and ${D}_{f}$ contour images of the surface were used, that were obtained by a modified “section method”, which is based on the fact that to obtain horizontal sections of irregularities located on the surface of digital twins, not a secant plane was used, but a linear measuring scale $l$, with which tracing was carried out of these irregularities in planes parallel to the base (*x;y*) along the minimum possible trajectories. At the first iteration, the value of the measuring scale ${l}_{m=1}$ was taken equal to the side of the studied square area ${l}_{1}=a$, and an automatic search and calculation of the number ${N}_{m=1}$ of the obtained non-intersecting closed contours of the first level $m=1$ was carried out. Further, the size ${l}_{1}$ was gradually decreased with the scan step $d=a/250$ to ${l}_{m=2}$ so that after tracing an integer number ${N}_{m=2} > {N}_{1}$ of non-intersecting contours of the second level $m=2$ nested in the contours of the first level was obtained, and so on until ${l}_{m}$ became equal to scan step ${l}_{M}=d$, where $m= 1, 2, 3, \dots , M$ is the level number, and $n=1, 2, 3,\dots , {N}_{m}$ is the number of contours at the *m*- th level. Thus, each contour is characterized by two numbers *n* and *m*. As a result, taking into account the total number of levels $M$, contours at each level ${N}_{m}$, the total number of contours in the system can be represented as $N={\sum }_{1}^{M}{N}_{m}$. In the process of tracing, the results were accumulated and a contour image of a digital copy on a plane was constructed, which was a set of non-intersecting contours of different levels nested into each other—the so-called contour image of the surface. In this case, each *m*-level corresponded to the measuring scale ${l}_{2}$, … ${l}_{m}$, … , ${l}_{M}$. Each of these closed contours is a section of a volumetric irregularity by a plane parallel to the base (*x;y*) and located at a certain height ${h}_{n}^{m}$ from it. It is clear that with a change in the measuring scale *l*_*m*_, the dimensions of the contours, their number ${n}_{m}$ and ${h}_{n}^{m}$ also change. In the case of fractal objects, a decrease in ${l}_{m}$ leads to an exponential increase in $N$ until the lateral sizes of irregularities in the (*x;y*) plane do not exceed ${l}_{M}=d$^31^.

To find these parameters, was used the Fractal analysis software package of the “NTEGRA SPECTRA” (version: “NOVA SPM 4.0”) atomic force microscope manufactured by “NT-MDT”, as well as the vector graphics package of the “CorelDRAW” (version: “Graphics Suite X8”) licensed software.

### Ethics declarations

This study was performed according to the principles of the Declaration of Helsinki. Approval for studies including collection of buccal epithelium cells was granted by the Ethics Committee of Sevastopol State University (Study No. 3; July, 15th 2021). Buccal epithelium cells were collected in accordance with the code of conduct of research with human material in the Russian Federation. All subjects gave written informed consent.

## Results and discussion

### The reaction of cell membrane to external micromechanical stimuli

For probe measurements of cells at the submicron and nanoscale levels and the choice of the force effect when scanning the membrane, our investigation will be focused on the description of the spectra of the force curves ${F}_{z}={F}_{z}(Z)$ of the main areas of the reference curves on approach and withdrawal. In the ‘setpoint’ parameter, the initial value of force action of probe ${F}_{const}= {F}_{z}({z}_{0}\equiv z=0)$ on the membrane at the point of its contact with the surface before carrying out the force measurements of AFM was set. The level of the initial force action corresponding to the zero coordinate along the “$Z$” axis ($z=0$).

In the beginning, was filmed the original force. The probe was retracted from the surface at a predetermined distance ${Z}_{up}\approx$ 500 nm and the approach curve ${F}_{z}={F}_{z}(Z\downarrow )$ was measured by forcibly lowering the probe to the lower point ${Z}_{doun}\approx$ − 300 nm. The withdrawal curve ${F}_{z}= {F}_{z}(Z\uparrow )$ up to the upper point ${Z}_{up}\approx$ 500 nm was measured. The reference spectra of the approach and withdrawal curves (Fig. 4) taken on the free silicon surface (Fig. 1a, point I) with known mechanical characteristics was used.

Let us consider the spectral characteristics of the approach process (Fig. 4, curve 1). The horizontal section *AB* describes the process of approaching the cantilever with the surface at a sufficiently large distance between them, in the absence of interaction, ${F}_{Z}=$ 0 (Fig. 4b, curve 1). The probe is influenced by Van der Waals force (${F}_{VW}$) as it approaches the surface. First, there are repulsive forces (${F}_{Z} >$ 0) ${F}_{Z}=+{F}_{VW1}(BC)$ ≈ 0.096 *μN*, due to a small potential barrier [${\varphi }_{b}\left(BC\right)$] = *eV* in the *BC* section. Second, forces of attraction arise (${F}_{Z}<$ 0) ${F}_{Z}=-{F}_{VW1}(CD)$≈ − 0.363 μN the potential barrier ${U}_{01}$ (section *CD*), which reach their maximum value in the immediate vicinity of the surface (point ${r}_{z0}$) (Fig. 4b, curve 1). Presumably the barrier ${\varphi }_{b}\left(BC\right)$ describes the process of overcoming the surface tension forces of the adsorption layer by the cantilever. The depth [${U}_{01}$] = eV of the potential barrier determines the maximum force of attraction of the probe to the surface and the work of adhesive forces. When the cantilever moves towards the surface beyond $D$ point, the repulsive forces ${F}_{H}>$ 0 ($H$—Hooke's law) begin to act on the probe, preventing its penetration into the near-surface region (Fig. 4a, section *DE*). In this case, despite the rather large value of elastic deformations $\Delta z>r$ and the presence of adhesive forces ${F}_{adh}$, the section DE on the attraction curve ${F}_{z}= F(z)\downarrow$ has an almost linear form with *K* ≈ Const. For this process $K=$ 8.3 ± 0.2 N/m. The DE segment is characterized by good linearity and the absence of hysteresis between the attraction and recoil curves (Fig. 4a, inset). This indicates that the adsorption layer on the silicon surface has practically no effect on the process of measuring its elastic characteristics.

Here it is convenient to introduce the concept of an effective surface-the z-coordinate (*D* point), at which repulsive forces arise.

When the cantilever approaches the surface beyond the point $E$, it leads to a critical increase in the force ${F}_{Z}>{F}_{H}$, violation of the linearity ${F}_{Z}={F}_{Z}(DE)$, and the appearance of plastic deformations ${\Delta h}_{stif}$. The growth of ${F}_{Z}$ promotes the transition of the probe-surface interaction into the region of destructive deformations and will not be considered in this work. These areas are schematically depicted on profiles A–A and B–B of cross-sections of the surface cell relief (Fig. 2).

The recoil curve (Fig. 4a, curve 2), in the general case, has similar characteristic sections. In particular, the linear dependence ${F}_{z}= F\left(z\right)\uparrow$ with $K\approx Const$ in the DE section practically coincides with the linear dependence of the recoil curve in this section. In general, the behavior of the recoil curve, depending on the state of the surface and the nature of the material, can significantly differ from the behavior of the attraction curve. The approach and withdrawal curves must match for uniform hard surfaces. The discrepancy between the approach and withdrawal curves (for example, large in modulus force of attraction $\left|{F}_{VW3}\right|>\left|-{F}_{VW2}\right|$, the absence of a barrier ${\varphi }_{b}$, for the bottom of a potential barrier ${U}_{02}$, an increase in coordinates), i.e. the hysteresis, indicates the features of the surface and near-surface region. The high coordinate of the potential barrier ${r}_{z02}>{r}_{z01}$, indicates the presence of an adsorption layer (adsorbate) with an effective thickness ${\Delta z}_{a}$ < 100 nm, on the surface at the point of contact with the probe. The presence of a thin nanometer layer of adsorbate in this case is caused by the remains of the buffer solution. According to the theory of capillary phenomena by T. Young, P. Laplace and J. Gibbs, the adsorption effect is largely determined by Van der Waals force^32–34^.

The coincidence of linear regions of both the approach and withdrawal curves in the *DE* region as well as the same slope ($\alpha$ angle) prove the homogeneity of the mechanical properties of epitaxial silicon layer near the surface, and indicate correct setup of the AFM instrument and the operation of cantilever. Thus, for the Si surface the shape of the approach and withdrawal curves fully corresponds to well-known physical model of the interaction of two atoms by means of Van der Waals force and located at a distance ***r***, from each other in accordance with the Lennard–Jones ${U}_{LD}$, potential^35^:

6 $${U}_{LD}\left(\mathbf{r}\right)={U}_{0}\left\{-2{\left(\frac{{\mathbf{r}}_{0}}{\mathrm{r}}\right)}^{6}+{\left(\frac{{\mathbf{r}}_{0}}{\mathbf{r}}\right)}^{12}\right\}.$$

Parameter ${\mathbf{r}}_{0}$ is the equilibrium distance between atoms, and ${U}_{0}$ is the minimum value of potential energy in the equilibrium system, i.e. the potential barrier bottom. The first term in this expression mainly describes the dipole–dipole attraction of atoms, and the second describes much shorter-range repulsion at short distances. The radius of action of Van der Waals force (≈ 10 nm) significantly exceeds the radius of overlap of wave functions of interacting nano-objects (≈ 1 nm). Van der Waals forces arise due to the fluctuation nature of the interacting electromagnetic forces between bodies: orientational, which is obtained as result of averaging over the equilibrium distribution of orientations of interacting dipoles; induction interaction (usually observed at elevated temperatures); dispersive quantum mechanical interaction. Note, the competition between Van der Waals forces of attraction and repulsion often takes an active part in the coagulation of colloidal systems, which in most cases include cell membranes.

Depending on the condition of surface and value of pressing force, it is possible to apply various mechanical influences on the membrane surface (approach curve) with the registration of the parameters of the membrane response (withdrawal curve) to external stimuli. The correct choice of initial value ${F}_{z}={F}_{const}$ of force during the AFM measurements of a biological object surface is a very important preparatory stage. For example, an excessively large ${F}_{const}$ value can lead to plastic deformations, or even rupture of the cell membrane, and an excessively small value can lead to excessive sensitivity of the method and the occurrence of interference associated in most cases with the presence of an adsorbate.

According to preliminary results, the cell membrane possesses good elastic properties and withstands elastic deformations along the $z$ axis significantly exceeding 300 nm with a force $F$>28 *nN* (Fig. 5).

These circumstances turned out to be very important, they made it possible not only to measure the membrane surface, but also to probe (accurately palpate) with non-destructive methods what is under it, and to significantly expand the range of force effects, excluding the possible influence of foreign objects on the surface. Elastic deformations commensurate with the thickness of cell testify the non-destructive action of probe (integrity, tightness of the membrane), which excludes the leakage of cell contents under study.

Relatively flat areas of the surface were selected (Fig. 1a, point II) with no organelles under them (Fig. 1a, indicated by arrows) were selected in order to define the optimal value of the constant pressing force ${F}_{const}$ for both contact and hybrid scanning modes. As a result, for the AFM measurements by the contact mode, the pressing force ${F}_{const}$ ≈ 18–22 nN was chosen, which provides not only a sufficient force effect, but also a good signal/noise ratio at a level of elastic deformations approx. 150 nm. Note that repeated scanning in more gentle modes (at ${F}_{const}$ < 5 nN) did not reveal any destructive effects from the cantilever.

As expected, the features of micromechanical properties of cell membrane were manifested in significant differences in the approach and withdrawal curves of force spectroscopy of the AFM method (Fig. 5).

First of all, it can be seen that the approach and withdrawal curves do not coincide in the *DE* sections, and the approach curves have a shorter length in comparison with the control curves. A larger slope ($\alpha$-angle) of linear sections of the approach curves indicates in this case a greater elasticity of cell membranes, i.e. a larger elastic coefficient $K$, which depends on the strength of initial action (pressing force ${F}_{const}$).

Figure 5a shows four cases differing in the ${F}_{const}$ value. In the case I at ${F}_{\mathrm{I},const1}$ = 4 µN the deformation was ${\Delta h}_{\mathrm{dfrm}1}$ = 69 nm and ${K}_{1}$ = 65.7 ± 0.2 N/m. In the case II with ${F}_{\mathrm{II},const1}$ = 6.2 µN, the deformation was ${\Delta h}_{\mathrm{dfrm}2}$ = 115 nm and ${K}_{2}$ = 67.4 ± 0.15 N/m. In the case III at ${F}_{\mathrm{III},const1}$ =10.1 µN, the deformation was ${\Delta h}_{\mathrm{dfrm}3}$ =220 nm and ${K}_{3}$ =80.2 ± 1.18 N/m. In the case IV at ${F}_{\mathrm{IV},const1}$ =21.6 µN the deformation was ${\Delta h}_{\mathrm{dfrm}4}$ = 321 nm and ${K}_{4}$ = 84.5 ± 0.22 N/m.

Index 1 corresponds to the approach curve. Moreover, in the first two cases (Fig. 5a, I and II) the region of deformations ${\Delta h}_{\mathrm{dfrm}}$ ≤ 115 nm falls on the elastic region, whereas in cases III and IV deformations ${\Delta h}_{\mathrm{dfrm}}$ > 115 nm, the deformation also affected the region of plastic deformation ${\Delta h}_{\mathrm{stif}}$.

Following the authors of ref.^10,18^, the coefficient of elasticity of the cell membrane was significantly higher than the coefficient of elasticity of the cantilever beam. Differences are also visible in the near-surface area of action of adhesive forces. First of all, attention is drawn to the absence of repulsive forces (barrier ${\varphi }_{b}(BC)$) and the so-called ‘beak’ (potential barrier ${U}_{0}$) on the approach curves, when approaching the adsorption layer (Fig. 4a, curves 1). This fact indicates a relatively low hydrophilicity of the membrane cell. In other words, micro- and nanoobjects approaching this point in the absence of a noticeable effect (pressing force *F*_*const*_) on the membrane will be very weakly held by its surface. On the one hand, it allows the cell moving more freely in the external environment (due to low wettability), and, on the other hand, to freely overcome the surface tension forces due to the lack of hydrophobicity. The withdrawal curves in all cases pass below the approach curves and have a quite complex dependence on the coordinate and, most likely, cannot be characterized by any coefficient of elasticity. A relatively small effect from an external nanoobject on a cell leads to an increase in the adhesive forces of attraction, which allows the cell to fix itself on external objects, or to retain on its surface the external micro- and nanoobjects leaving it.

Case I of the withdrawal curves have more in common with the withdrawal test curves (Fig. 3). They also have a linear section, a potential barrier region ${U}_{0}$ and a neutral region of no interaction. On the surface of cell membrane rather thick adsorption buffer layer ${\Delta z}_{a}$ < 100 nm (Fig. 5b*,* ${\Delta z}_{a}$) was noted, which significantly exceeded the adsorption layer on the free surface of the silicon substrate, according to the behavior of the withdrawal curves. This layer protected the cells from complete drying out for a sufficient (3–4 h) period of time for measurements. Such conditions for AFM scanning of cell surface are intermediate between measurements of a completely dried (dry) non-living cell (for example, Fig. 1b) and measurements of living cells in liquid medium. The mixed scanning mode for the AFM measurements of biological objects is easier to use and to some extent combines the advantages of classic ‘dry’ and ‘wet’ scanning modes. Similar to case II in Fig. 5a, the behavior of both approach and withdrawal curves when studying elasticity of the local 20 × 20 µm regions of human intestinal Caco-2 cell membranes by means atomic force spectroscopy was observed by the authors of refs.^3,12^, but no explanation was given.

Given the adsorption layer ${\Delta z}_{a}$ the *F*_*Z*_ value should be increased experimentally by the ${+F}_{VW1}(BC)$ sufficient for probe to penetrate (Fig. 4b). As the thickness ${\Delta z}_{a}$ and the homogeneity of the adsorption layer may not be the same everywhere, the increase in the ${F}_{z}$ value should be accomplished with a margin falling down into the *DE* region of elastic or plastic deformations.

It was found that the reaction of the membrane of the living human buccal epithelium cell to an external micromechanical stimulus which is the level of the initial force effect ${F}_{const}$ is not a simple mechanical process, but is nontrivial one depending on the level of passive or active stimulation (force of influence). It was found that, by analogy with external active and passive stimulation, a human buccal epithelium cell can exhibit both an active and a passive reaction of its membrane to an external micromechanical stimulus.

The first passive type includes responses due to the elastic mechanical properties of membrane. This level of action for cells did not exceed ≤ 6 nN, was accompanied by the appearance of elastic deformations on the membrane and did not require the consumption of internal energy resources of cell to restore its original shape. In this case, the linear sections on the approach and withdrawal curves coincided, and on the withdrawal curve in the immediate vicinity of surface a ‘beak’ ${F}_{VW3}$ ≈ − 0.2 µN (potential barrier) appeared, i.e. the adhesive forces of attraction holding the probe on the membrane surface (Fig. 5a, I and II).

The second active type of response reactions arose at stronger initial load 6 < ${F}_{\mathrm{III},const1}$ < 21 nN and already required an increased consumption of energy resources. In this case, the approach and withdrawal curves no longer coincided, i.e. the initiation of hysteresis had begun and the appearance of nonlinear sections indicating the presence of plastic deformations (Fig. 5a, case III). An increase in the level of initial force action ${F}_{const}$ leads, as a rule, to the bending of nonlinear section upwards. Thus, plastic deformations required increasing external force effects with an increase in the size of deformation (the approach curve went up from the linear dependence—the dotted line). It means that in this type of response the cell membrane actively increases its resistance to external micromechanical influences during the transition to plastic deformations. In such case when the probe moves upward from the surface, a ‘beak’ ${F}_{\mathrm{VW}3}$ ≈ − 0.16 µN (potential barrier) is also observed on the withdrawal curve in the immediate vicinity of the surface. At the same time, the degeneration of the initial value ${F}_{\mathrm{III},const}$ into ${F}_{\mathrm{III},const1}$ and ${F}_{\mathrm{III},const2}$ begins (Fig. 5a, the inset). The withdrawal curve in this case is slightly lower, but in relative proximity to the approach curve. For the cell membrane it requires a minimum of energy to restore its original state.

The third passive type of response arose with a further increase in the level of active stimulation of cell over 21 nN and was accompanied by passive resistance (decreasing with an increase in external exposure) of membrane to external exposure and significant plastic deformations (Fig. 5a, IV). In contrast to the previous case, plastic deformations now, on the contrary, require less force effects relative to the linear section (the approach curve deviates downward from the linear dependence—the dotted line (Fig. 5a, IV)). This means that the cell membrane at a certain level of force (${F}_{const}$ ≈12 nN) changes its response to it–it stops resisting, begins to more actively succumb to external force and deform plastically. At the same time, the degeneration of the initial value of ${F}_{\mathrm{IV},const}$ increased in ${F}_{\mathrm{IV},const1}$ and ${F}_{\mathrm{IV},const2}$ (Fig. 5a, diagram), and stronger differences were observed in the behavior of the approach and withdrawal curves.

In the second and third types of responses, despite the presence of plastic deformations, after gradual removal of the load (external influence) the cell membrane begins to independently restore its shape, starting from a certain level of external influence (for each load, this level is different). This requires the consumption of internal energy and information resources of cell. This, in particular, is indicated by a noticeable hysteresis caused by the difference in the elastic properties of the initial and plastically deformed areas of the surface membrane. According to the linear section of the withdrawal curve, the plastically deformed region upon gradual removal of the load behaves like an elastic medium with its own coefficient of elasticity (Fig. 5a, IV).

In a mechanical process gradual stress relief would bring the withdrawal curve to the ${Z}_{\mathrm{deform}}$ point defining the size of the plastic deformation ${\Delta h}_{\mathrm{stif}}=\Delta z-{Z}_{\mathrm{deform}}$. As follows from the behavior of the withdrawal curve, the area of plastic deformation at ${F}_{\mathrm{IV},\mathrm{const}}$ ≈ 5 *nN* begins to restore its mechanical properties, and the withdrawal curve returns to its original trajectory. Such membrane behavior can no longer be reduced to a simple mechanical process. At a certain moment, the cell membrane using information and energy resources, begins to do the work to restore its shape. The width of the ‘beak’ ${\Delta z}_{\mathrm{a}}$ ≈ 100 nm, in this case remains practically unchanged (Fig. 5b).

Thus, the reaction of cell membrane to the action of probe is not a simple mechanical process but, depending on the mode of action, is a selective, non-trivial, ‘individual’ process.

### Surface relief of the cell membrane

**Figure 6:**
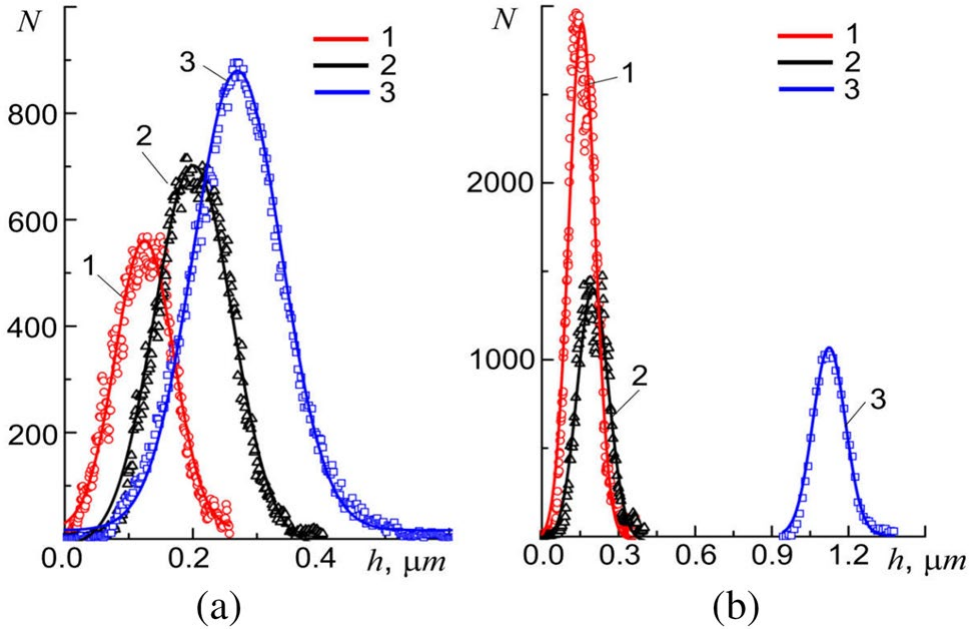
Histograms $N=N(h)$ of distribution of relief unevenness of square section of the cell membrane surface, depending on the measurement method in the local approximation (**a**) at constant resolution 256 × 256 pixels, but different linear dimensions: 1—5 × 5 µm, 2—10 × 10 µm, and 3—20 × 20 µm (points—experiment, lines—Gaussian approximation); (**b**) at constant size 10 × 10 µm, but different resolution: 1—512 × 512 pixels, 2—256 × 256 pixels, and 3—128 × 128 pixels (points—experiment, lines–Gaussian approximation).

For detailing, a rectangular section of the surface of membrane limited by dimensions 10 × 10 µm (Fig. 2a, dotted square) will be further considered. According to the results obtained, the surface membrane of human buccal epithelium cell is not smooth but has a sufficiently developed Brownian relief, $h\left(x;y\right)$, formed by randomly spaced folds and other irregularities up to 500 nm in height. Statistical analysis revealed a non-trivial dependence of parameters values of the relief $h\left(x;y\right)$ on the methods of its measurement (Fig. 6). The main statistical parameters of the histograms of distribution of irregularities $N=N(h)$ of relief: the average value, $\langle h\rangle$, the variance, $\sigma$, and the standard deviation, $\sqrt{{\sigma }^{2}}$ (standard spread), completely depend on the size of measuring $l$ scale and the methods of obtaining it.

An increase in the linear measuring $l$ scale due to an increase in dimension of a square section from 5 × 5 µm to 20 × 20 µm at a constant resolution of 256 × 256 points (pixels) lead to an increase in the values of these parameters (Fig. 6a). An increase in $l$ due to increase in the number of scanning points (pixels) from 128 to 512 with constant linear dimensions of 10 × 10 µm of the area, on the contrary, leads to decrease in the values of these parameters (Fig. 6b). A similar behavior is observed for self-affine objects with fractal properties in the local approximation in a certain range of measurement scales^31^. In the global approximation (classical case) an increase in the size of measuring scale leads to an increase in the variance and a decrease with constant mean values.

**Figure 7:**
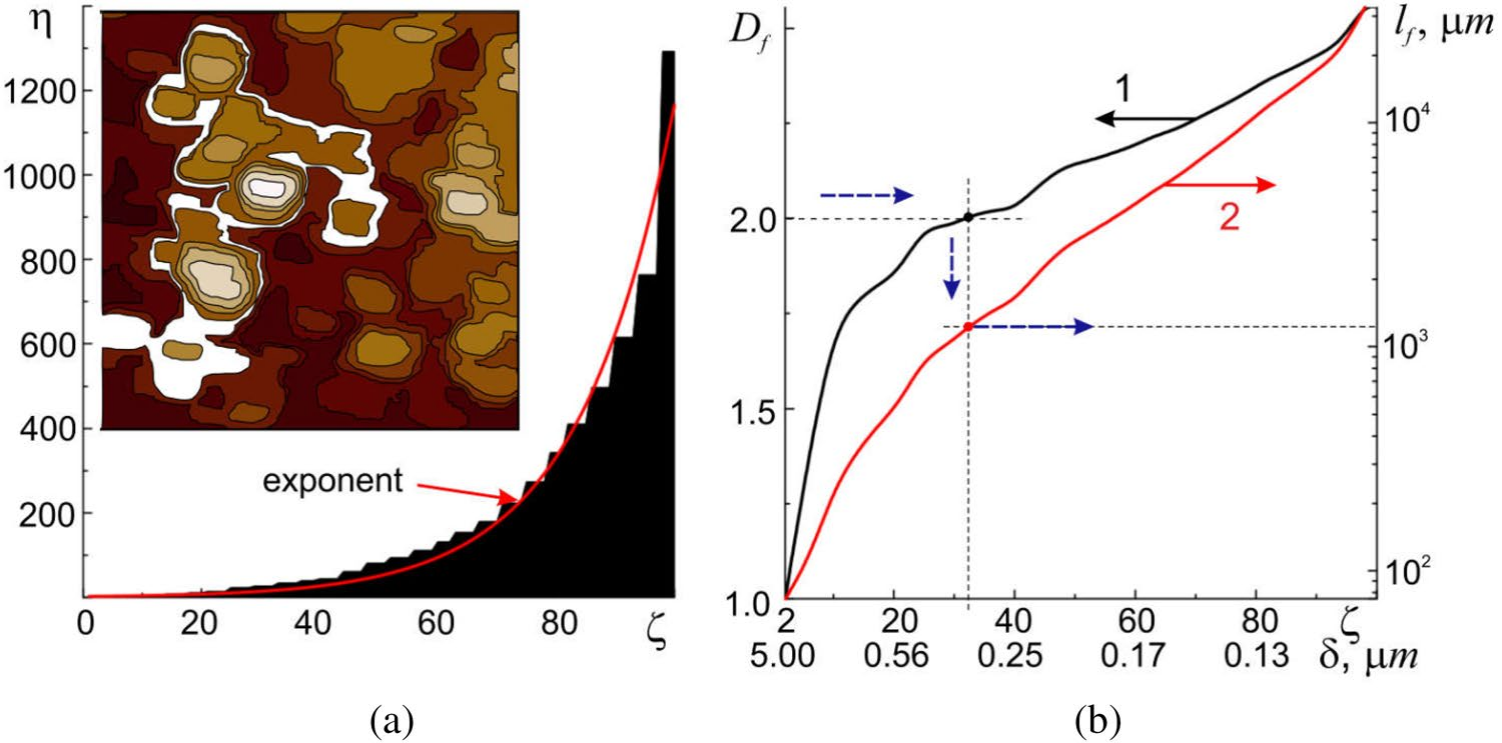
Graphical representation of ‘damn’ staircase, $\eta =\eta (\zeta )$, set of 10 × 10 µm contour image of the investigated area of cell membrane surface (**a**). Dependences of fractal dimension, ${D}_{f}={D}_{f}(\zeta )$–curve 1, and the actual length of the traveled path, ${l}_{f}={l}_{f}(\zeta )$—curve 2, depending on the values of the scaling coefficient,$\zeta$. The values $\zeta$ are labeled with the corresponding values for the area under study, $\delta$, in µm (**b**).

To answer the question what is the geometric surface relief of the cell membrane, the apparatus of fractal geometry (see the subsection "Fractal surface analysis method") will be used. Let us carry out the contour tracing of the surface relief irregularities and calculate the change in the number of the obtained contours depending on the change in the dimensions of measuring scale (Fig. 7a, inset). Changing $l$ $\zeta$ times we get $\eta$ contours inscribed into each other that satisfy the rule of topological mixing, i.e. the contours do not intersect with each other. A strong dependence on the initial conditions (the state of cell and the method of sample preparation), the dependence of the system parameters on the point view of an external observer ($l$ dimensions), and the property of topological mixing are necessary and sufficient signs of chaotic system, i.e. the surface relief. The mathematical apparatus of fractal geometry is now successfully used to describe such systems^31^.

As mentioned above, $\zeta$ and $\eta$ are important parameters of fractal (self-similar) objects and are called scaling coefficients. For ideal fractal objects (e.g. a triangle, or a Sierpinski napkin), the quantities $\zeta$ and $\eta$ have constant values independent of $\delta$. For real objects, as in our case, the quantities $\zeta$ and $\eta$ usually have different values in different intervals of measurement scales, which indicates a complex multifractal geometry of the surface relief. Due to the different values of the step sizes, the graphical representation $\eta =\eta (\zeta )$ is called the ‘devil's’ staircase (Fig. 7a). According to Ref.^31^, the quantities $\zeta$ and $\eta$ determine the value of the similarity dimension ${D}_{s}= \mathrm{ln}\left(\zeta \right)/\mathrm{ln}(\eta )$, which is directly related to the fractal dimension ${D}_{f}={D}_{T}+{D}_{s}$.

Having the values $\eta$, $\zeta$ and ${D}_{f}$ from Eq. (7), one can find the effective (real) values of geometric parameters as, e.g., the real surface area ${S}_{surf}$ or the length *l*_*f*_ of the path traveled over the membrane surface in each specific case:

7 $${S}_{fact}={S}_{0}{\left[\frac{{l}_{i}}{{l}_{0}}\right]}^{{D}_{T}-{D}_{f}}, {l}_{f}={l}_{0}{\left[\frac{{l}_{i}}{{l}_{0}}\right]}^{{D}_{T}-{D}_{f}}.$$

For example, the actual area of the upper membrane surface ${S}_{fact}$ for $\delta$ = 1/100 is 63,913.44 µm ^2^, which is more than 13 times greater than the area of its projection (${S}_{1}$ ≈ 4848.33 µm^2^).

**Figure 8:**
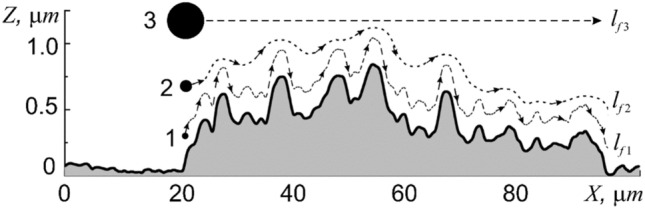
Section A-A of the relief of human buccal epithelium cell and a schematic representation of the traveled path of different sizes particles: 1—100 nm in diameter, 2—320 nm in diameter, and 3—5 µm in diameter.

The dependence of scaling coefficients on the measurement scale *l* leads to the multifractal dependence ${D}_{f}={D}_{f}({l}_{f})$, characteristic of real objects, which, according to Fig. 7b (curve 1), has values in the range from 1 to 2.6. An important point in the dependence ${D}_{f}={D}_{f}({l}_{f})$ is the point with ${D}_{f}$ = 2 corresponding to the value $l$ = 320 nm. In particular, it follows that an external object (for example, a particle) with a size of approx. 320 nm will not feel irregularities less than 320 nm, and the membrane will be perceived as a two-dimensional surface with ${D}_{f}={D}_{T}=$ 2 (Fig. 8, particle 2).

An increase in the size of the external object to $l$ = 5 µm and more will lead to the case when the value of ${D}_{f}$ will tend to 1 (Fig. 8, particle 3). In other words, such particles will not feel the roughness of the surface relief at all (for them the surface will be ideally smooth) and can cross it along trajectories that are straight lines. Objects less than 320 nm will feel the finer surface irregularities and move along the membrane as if on a three-dimensional fractal surface, which will lead to a significant increase in their path (Fig. 8, particle 1).

For instance, several trajectories of motion of particles of different sizes along the cross-sectional surface of cell A–A in Fig. 8 (dotted paths) will be considered. The section projection onto the base has a length ${l}_{1}$ = 75 μm. Using the expression (5) in which ${M}_{1}={l}_{1}$ is the projection of the cross-section of the relief onto the base, it is possible to estimate the actual path that the particle needs to travel from one edge to the other. A particle of 5 µm in size (${D}_{T}={D}_{f}$ = 1) will cross the cell along the shortest path, the length of which completely coincides with the projection length ${l}_{f3}$ = ${l}_{1}$ ≈75 µm. A particle of 320 nm (${D}_{T}={D}_{f}$ = 2) will cover a path of length ${l}_{f2}$ ≈ 349.3 µm, and a particle of 100 nm (${D}_{T}={D}_{f}$= 2.56) will cover ${l}_{f1}$ ≈ 988.7 µm. With other things being equal, smaller particles will have to travel longer distances on the surface of cell membrane, which will significantly limit their transport capabilities.

**Figure 9:**
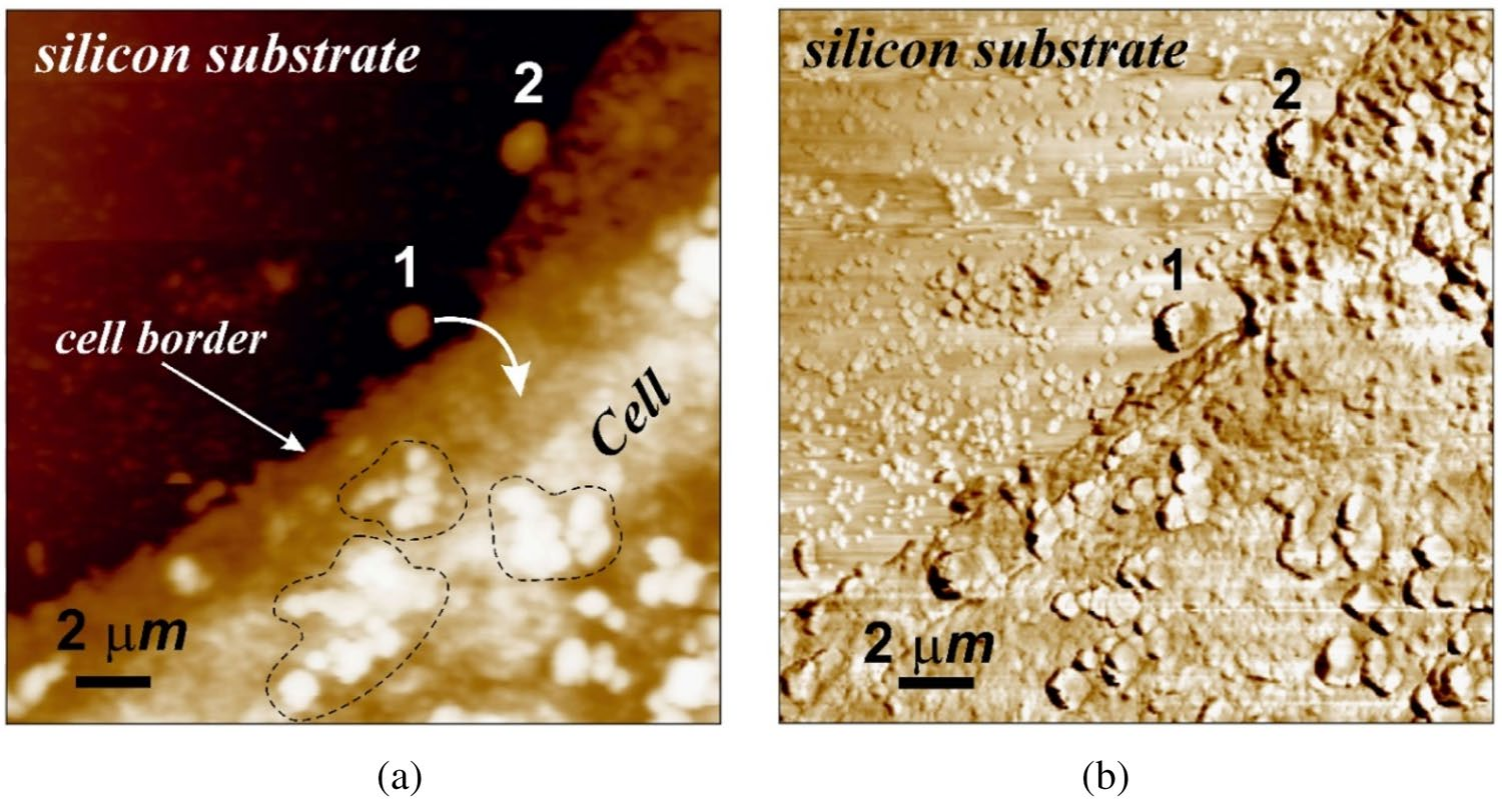
AFM images of a 20 × 20 µm area of the surface of human buccal epithelium cell edge on a silicon substrate with micro- and nanoparticles adsorbed on them, (**a**), and its phase contrast, (**b**).

Experimental studies of the movement of particles across the surface of cell membrane confirm these conclusions. Figure 9 shows an image of a human buccal epithelium cell edge adjacent to the surface of a silicon substrate containing a large amount of adsorbed micro- (< 2 µm) and nano- (> 30 nm) particles. The observed nano- and microparticles were formed on a silicon substrate during drying of the buffer solution. The study of the phase composition of the surface of the steam showed that the original surface of the cell membrane was free of such particles. As follows from this figure, the largest over 1 µm particles (e.g. 1 and 2, Fig. 9a) are the first to reach the cell surface and begin to move along it. This is evidenced by the increased concentration of such particles on the membrane surface, which is the concentration gradient with respect to the silicon substrate. There are practically no particles on the substrate (with the exception of particles 1 and 2 in Fig. 9a) with particles larger than one micrometer; all of them are not only located on the membrane surface, but also managed to form rather large clusters (Fig. 9, circled areas). These conclusions are in good agreement with the phase contrast image for almost all nanoparticles less than approx. 400 nm in size located on the surface of the silicon substrate, while the larger ones are almost all located on the cell membrane (Fig. 9b). In particular, while particle 1 with approx. 1 µm in size is approaching the membrane surface, another similar particle 2 has already reached its surface.

Analysis of the AFM images showed that the movement of the observed particles over the membrane surface is not accompanied by its plastic deformations along their trajectories. Below it will be shown that the force required for the appearance of membrane plastic deformations is much higher than the weight of the observed micro- and nanoparticles. Thus, the fractal parameters of the surface relief of the cell membrane are necessary to describe the mechanisms of interaction of nano-objects with the surface, the distance traveled, the speed of movement, the parameters of energy dissipation, the mechanical work performed in this case, thermodynamic potentials, etc.

### Mapping the micromechanical properties of the membrane of buccal epithelium cell

Investigation of the architecture of cell membranes and their micromechanical properties requires complex mapping of their functional characteristics with the necessary resolution both in absolute value and in spatial coordinates. As such functional characteristics (hereinafter—the characteristics) one can consider micromechanical characteristics (elasticity (Young's modulus $E=E(x;y)$), elastic ${\Delta h}_{dfrm}$ and plastic ${\Delta h}_{stif}$ deformations) of cell membranes. For the process of measuring the parameters of these characteristics, a point or grid method of local research with a very limited resolution is often used, e.g. 16 × 16 points (pixels)^36^ aimed at collecting statistical data. In this case, the sizes of local areas usually do not exceed a few micrometers, which makes it difficult to study the architecture of outer exoskeleton of cell membrane and its micromechanical properties, as well as the relationship between the morphology of the membrane surface and its micromechanical characteristics. Currently, there are no general guidelines for choosing the required scanning conditions. Everything is determined by the capabilities of the AFM, the requirements of the researcher, the size and condition of the cell, and external conditions.

The human buccal epithelium cells belong to rather large biological micro-objects, i.e. adult eukaryotes can reach hundreds of micrometers, with a sufficiently developed landscape of the membranes surface. In our opinion, to obtain satisfactory mapping results for complex functional characteristics of such objects, the optimal sizes of local sections of the membrane surface should be at least 10 × 10 µm with a minimum resolution of 100 × 100 scanning points in frame and line scans.

According to subsection "AFM methods", in order to solve the problem of mapping by AFM methods a hybrid method was used, which makes it possible to simultaneously obtain data on the relief and the above-mentioned micromechanical properties of the surface at each scanning point. For the purpose of reproducible data measurement and recording the approach and withdrawal curves at each point, an extended range of external force effects from 0 to 40 µN was used with a sufficiently high level of initial force action ${F}_{const}$ ≈ 21 µN significantly exceeding the level required for the occurrence of elastic deformations ${\Delta h}_{dfrm}$. The case of destructive deformations is not considered in this work.

**Figure 10:**
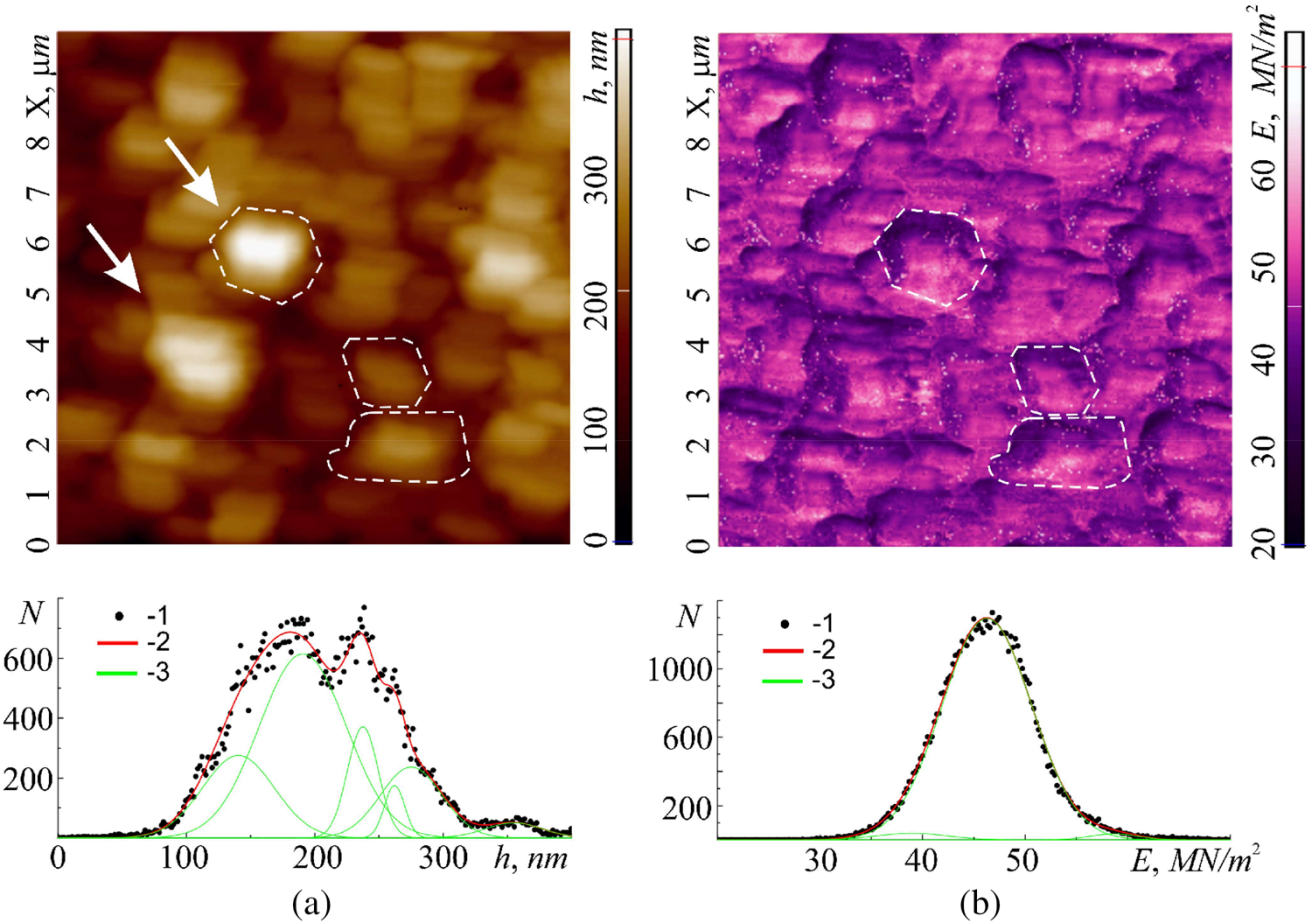
Image of a 10 × 10 µm cell surface area obtained in the hybrid AFM mode at a resolution of 100 × 100 scanning points: relief $h=h(x;y)$ (**a**) and Young's modulus $E= E(x;y)$ (**b**) with their histograms $N=N(h)$ – 2 and $N=N(E)$ – 2, respectively. Points (1)—experiment, curves (3)—approximation by Gaussian functions. Elements of the mesh structure are outlined with dotted line.

**Figure 11:**
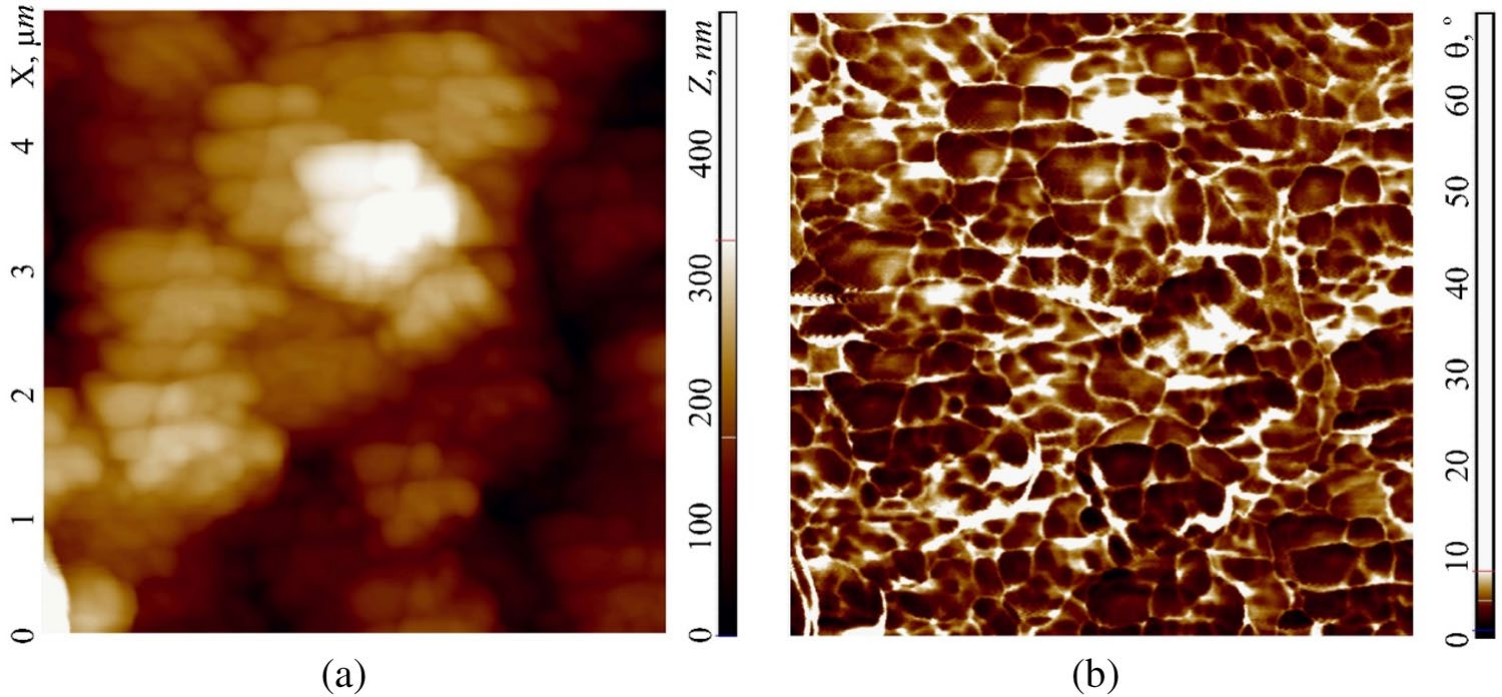
5 × 5 µm image of cell membrane surface area obtained in the semicontact AFM mode at resolution of 500 × 500 scanning points: relief $h=h(x;y)$ (**a**) and phase contrast $\theta = \theta (x;y)$ (**b**).

As reported in subsection "Surface relief of the cell membrane", the membrane of human buccal epithelium cells is not a smooth surface, but has a self-affine multifractal Brownian relief with an average vertical size of irregularities (tubercles) up to 500 nm. In the horizontal plane, the surface has a rather large cellular structure with sizes from 200 to 2000 nm (Fig. 10a, highlighted by a dotted line). The size of the irregularities depends on the cell age and its condition. More detailed studies of cells (in semicontact scanning AFM mode at the higher resolution of 500–500 points), in its turn, revealed their grid structure with sizes from 50 to 350 nm (Fig. 11). The mesh construction of the exoskeleton at the nanoscale is very widespread among cell membranes (for instance, see Ref.^37^). In this case it clearly manifests its fractal geometry not only at the submicron level, but also at the micron level. Of interest are the size and shape of such cells, which, according to the available data, can be ‘bags’ of a disordered porous peptidoglycan gel, which is a three-dimensional network in a liquid medium^38^. This assumption is supported by data on the study of the process of coagulation of peptidoglycan and destruction of the mosaic structure of buccal epithelium of non-living cells. During long-term (> 20 min and temperature approx. 40 °C) air drying, the protective adhesive layer of the buffer solution disappears from the cells surface. After that, coagulation of its protein begins, which manifests itself in the destruction of the original structure and the formation of peptidoglycan dendrites on the membrane instead of it, the structure of which is probably similar to the structure of the peptidoglycan molecules themselves (Fig. 2b).

Statistical analysis indicates that the histogram of the membrane relief roughness $N= N(h)$ is formed by six Gaussians. This indicates that the felt unevenness in the relief corresponds to six different physical objects. To identify the correspondence of each of the six Gaussians to its object, it is necessary to consider the topography of the remaining functional characteristics.

The topographic image of the elastic parameters (Young's modulus $E$) of the considered surface area (Fig. 10b, highlighted by dotted line) also revealed a large cellular structure. Despite the fact that the surface can be characterized by an average value of $E$ ≈48 × 10^6^ N/m^2^, each cell translates areas with increased and decreased elasticity. Cell boundaries also have separate elastic properties. This is fully confirmed by the statistical analysis of the surface topography, $E= E(x;y)$. The histogram of the distribution $N= N(E)$ in this case is formed by three Gaussians, each of which describes the elasticity of its local area in the cell: one Gaussian corresponds to the boundaries of the mosaic structure, the second to local areas with increased elastic properties, and the third to those with the reduced ones. On the raster image of the topography $E=E(x;y)$ the analysis of the intensity of monochrome shades makes it possible to determine the mutual correspondence of Gaussians to local areas with different elasticities. The average value of fractal dimension $E=E(x;y)$ was $\langle {D}_{f}(E)\rangle$ =2.61, obtained by the triangulation method.

**Figure 12:**
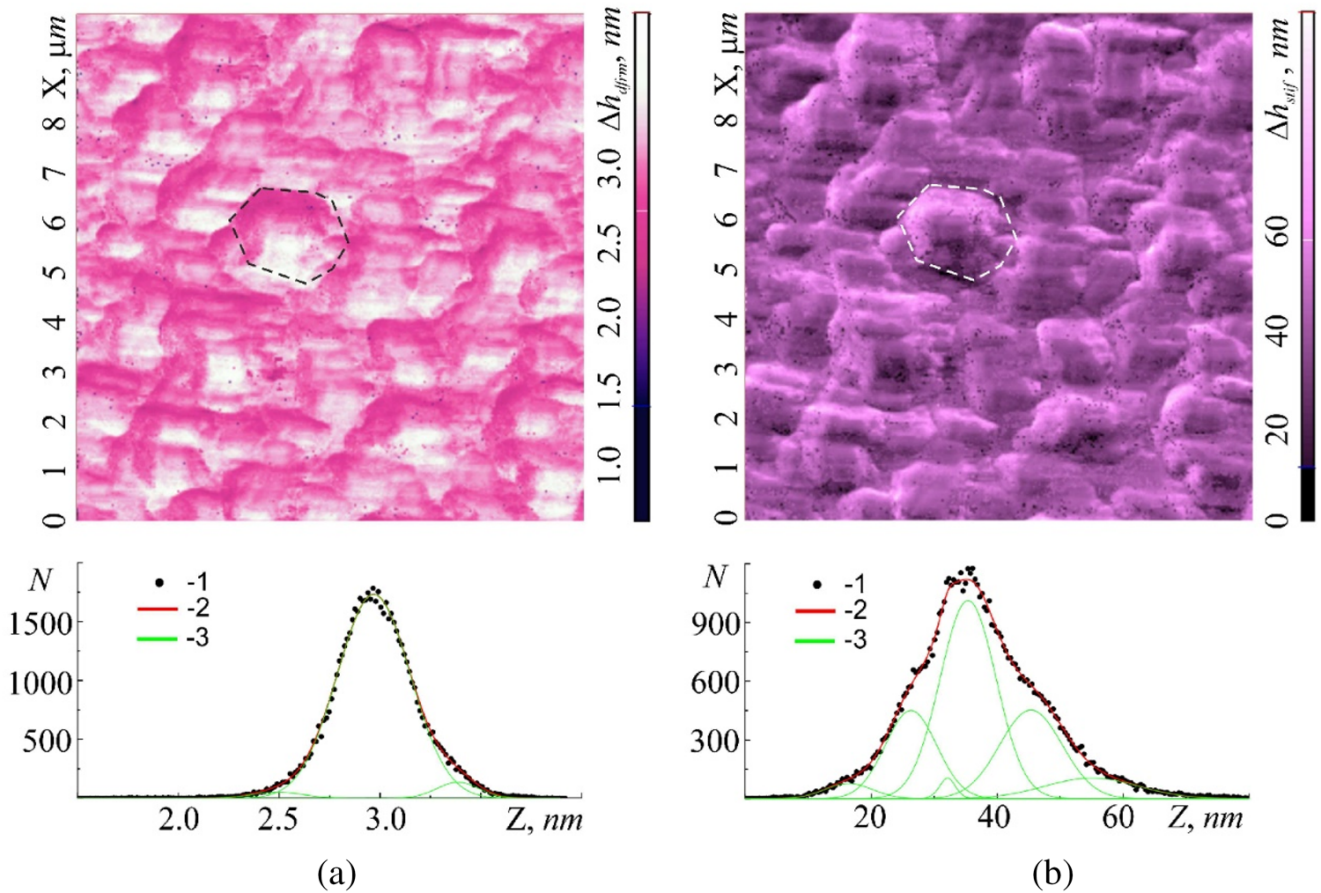
Topographic images of 10 × 10 µm section of the cell membrane surface obtained in the hybrid AFM mode at a resolution of 100 × 100 scanning points: elastic deformation ${\Delta h}_{dfrm}={\Delta h}_{dfrm}(x;y)$ (**a**) and plastic deformation, ${\Delta h}_{stif}={\Delta h}_{stif}\left(x;y\right)$ (**b**) with their own histograms, $N=N({\Delta h}_{dfrm})$ and $N=N({\Delta h}_{stif})$—(2), respectively. Points (1)—experiment, curves (3)—approximation by Gaussian functions. The dotted line marks the borders of cell.

Simultaneously with Young's modulus *E* it is convenient to consider the level of elastic ${\Delta h}_{dfrm}$ and plastic ${\Delta h}_{stif}$ deformations in this area. In both cases, the deformations reflect the coarse structure of the cell membrane. The amplitude of elastic deformations ${\Delta h}_{dfrm}$ along the ‘$Z$’ axis can exceed 4 nm. The image contrast ${\Delta h}_{dfrm}={\Delta h}_{dfrm}(x;y)$ fully corresponds to the image contrast $E=E(x;y)$ (light local areas in Fig. 10b with large values of $E=E(x;y)$ correspond to light areas (large elastic deformations ${\Delta h}_{dfrm}$) in the image ${\Delta h}_{dfrm}= {\Delta h}_{dfrm}(x;y)$ (Fig. 10a)). The dark areas in Fig. 10b with small values of $E=E(x;y)$ correspond to dark areas ${\Delta h}_{dfrm}={\Delta h}_{dfrm}(x;y)$ in Fig. 12a with small deformations. The location of the boundaries of local regions of elastic deformations practically corresponds to the location of the cell boundaries in the relief image (Fig. 10a). The results of the statistical analysis ${\Delta h}_{dfrm}={\Delta h}_{dfrm}(x;y)$, correlate with the results of a similar analysis $E=E(x;y)$. The histogram $N=N({\Delta h}_{dfrm})$ as well as the histogram $N=N(E)$ is formed by three Gaussians, which describe the normal distribution of deformable regions, as well as their boundaries. Small values of ${\Delta h}_{dfrm}$ indicate that only thin surface layer subjected to elastic deformations, which do not affect the deep-lying layers of the cell membrane and inside the cell. The average value of the fractal dimension $\langle {D}_{f}({\Delta h}_{dfrm})\rangle$ was 2.68, obtained by the triangulation method $\langle {D}_{f}({\Delta h}_{dfrm})\rangle$.

The amplitudes of plastic deformations ${\Delta h}_{stiff}$ significantly exceed the amplitudes of elastic ones and can reach over 100 nm along the $z$-axis (Fig. 12b). Moreover, each cell contains regions with both small and large plastic deformations. Comparing Fig. 12a and b one concludes that the most elastic regions of cells (light regions) are subjected to smaller plastic deformations (dark regions), and less elastic regions (dark regions) are subjected to large plastic deformations (light regions), which fully corresponds to classical theory of deformation of macroscopic objects.

Comparison of the histograms reveals significant differences in the methods of manifestation of elastic and plastic deformations. The histograms of both the distribution of plastic deformations $N=N({\Delta h}_{stif})$ and the relief $N=N(h)$ are formed by six Gaussians. Due to the large values of ${\Delta h}_{stif}$ they describe not only plastic deformations of the outer, but also of the inner layer of membrane as well as the inner content cells, i.e. the organelles underneath. This fact significantly expands the capabilities of AFM in the direction of studying not only the surface, but also the bulk properties of living cells.

After removing the load during repeated scanning, the cell membrane almost completely restores its original shape. Again, this confirms the results of similar studies described in subsection "The reaction of cell membrane to external micromechanical stimuli" on restoring the original shape of the approach curve. The average value of the fractal dimension $\langle {D}_{f}({\Delta h}_{stif})\rangle$ was 2.42, determined by the triangulation method.

Analyzing the histograms $N=N(E)$, $N=N({\Delta h}_{dfrm})$ and $N=N({\Delta h}_{stif})$, it can be concluded that three of five Gaussians $N=N(h)$ describe the normal statistics of the cellular structure regions with increased and decreased elastic properties, as well as connective tissue that holds together individual cells (boundaries).

Comparing the raster images of the reliefs in Figs. 2a and 10a, we can say that the shape of the two prominent irregularities indicated by arrows in Fig. 10a can be connected by the organelles located under them. Thus, the next two Gaussians describe the normal statistics of plastic deformations under the membrane of two organelles and, according to Fig. 12b, having the highest level of plastic deformations in the investigated area.

**Figure 13:**
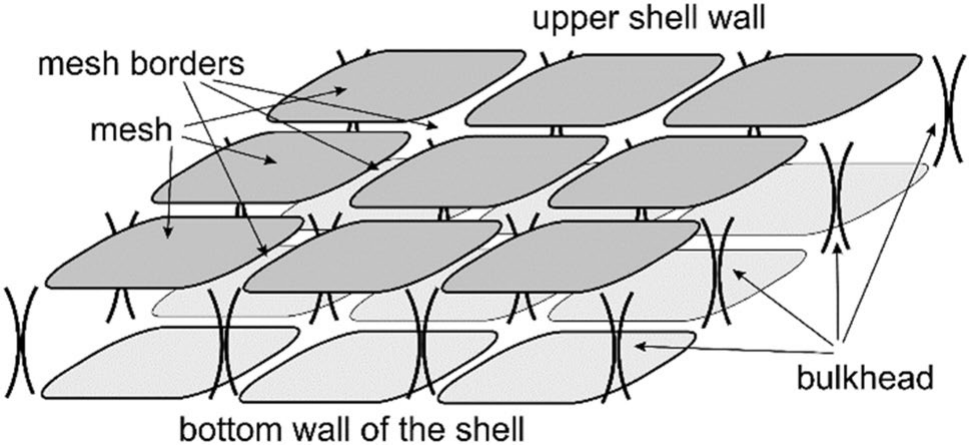
Schematic representation of some nodes of the mechanical structure of cell membrane of the human buccal epithelium cells.

The investigated buccal epithelial cell has a very flat shape approx. 75 µm in width and approx. 100 µm in length with a maximum thickness of less than 1.5 µm. From general physical considerations, it follows that in order to maintain such a shape, a cell must have not only a complex external mechanical structure (an exoskeleton), but also an internal one (bulkheads or bridges) that hold the upper and lower walls of cell membrane at a certain distance from each other (Fig. 13, bulkhead). The fifth Gaussian $N=N({\Delta h}_{stif})$ can describe the normal distribution of plastic deformations of local attachment places of bridges with membrane walls.

Thus, the results obtained indicate not a sandwich-type (according to which the lipid bilayer is located inside, the inner and outer sides of which are covered with continuous layers of surface proteins), but a more complex helium-mosaic structure of the envelope of human buccal epithelium cell.

## Conclusion

The results obtained in the work confirm the fulfillment of the set goal of studying the reaction of cell membranes of living adult cells of the human buccal epithelium to external micromechanical stimuli in a wide range (up to 40 *μN)* of force effects: the relief geometry $h(x;y)$ and mapping of its micromechanical (Young's modulus $E=E(x;y)$, elastic ${\Delta h}_{dfrm}= {\Delta h}_{dfrm}(x;y)$ and plastic ${\Delta h}_{stiff}={\Delta h}_{stiff}(x;y)$ deformations), as well as adhesive characteristics (work $A=A(x;y)$ of adhesive forces ${F}_{adh}={F}_{adh}(x;y)$).

The use of a thick protective adhesive layer of < 100 nm buffer solution, proposed by the authors for the first time, on the surface of these cells during scanning showed its effectiveness, as it prevents cell death from rapid drying and allows one to use the advantages of both dry and wet methods of scanning living biological objects in atmospheric air under normal conditions for a sufficiently long period of time (in this case more than 3 h).

The justification for the use of the MDT (Derjagin–Muller–Toropov) model of the interaction of the probe with the surface of the cell membrane in the contact and hybrid scanning modes is confirmed by the large deformations of the membrane that occur during scanning in the used range of force effects, significantly exceeding the radius of the needle tip *r*. The use of the MDT model allows, during scanning, the simultaneous measurement of not only geometric—the shape of the relief $h=h(x;y)$, but also the functional micromechanical characteristics of cell membranes, taking into account adhesive forces. This approach makes it possible to link the change in membrane morphology with its functional characteristics at a new quantitative level. The large thickness of the adsorption layer makes it possible to exclude the influence of capillary effects on the process of measuring the work $A=A(x;y)$ of adhesive forces ${F}_{adh}={F}_{adh}(x;y)$ and mapping them.

The adaptation of the mathematical apparatus of fractal geometry to describe functional characteristics allows one to quantitatively study and model size effects not only in geometric, but also in micromechanical and adhesive functional characteristics of human buccal epithelium cell membranes, and also to extend it to other biological objects.

For the first time, it was found that in the local approximation at the micro- and nano-levels, almost all geometric and functional characteristics of the membrane are affected by the so-called size effect—the dependence of the measured measures on the dimensions of the measuring scale, and obey the mathematical laws of fractal geometry.

Thus, the surface of the outer shell of living adult cells of the human buccal epithelium is not smooth, but has a self-affine multifractal Brownian relief with a dimension of ${D}_{f}$ < 2.56 and an irregularity size of < 500 nm vertically and < 2 μm horizontally. It was shown that the fractal parameters of the cell membrane surface relief *ζ*, *η* and ${D}_{f}$ are necessary to describe the size effects arising from the interaction of nano-objects with the surface: the distance traveled, the speed of movement, the parameters of energy dissipation, the mechanical work done, thermodynamic potentials and so on. So, for instance, the actual area of the upper surface of the membrane ${S}_{fact}(\delta =1/100)$ = 63,913.44 μm^2^ is more than 13 times the area of its projection onto the substrate (${S}_{1}$ ≈ 4848.33 μm^2^), which, for example, must be taken into account when determining the surface concentration, or surface energy density.

The study and mapping of functional micromechanical and adhesion properties of the cell membrane surface (Young's modulus $E=E(x;y)$*,* ${D}_{f}$ = 2.56; elastic ${\Delta h}_{dfrm}={\Delta h}_{dfrm}(x;y)$, ${D}_{f}$ = 2.68; and plastic ${\Delta h}_{stiff}={\Delta h}_{stiff}(x;y)$, ${D}_{f}$= 2.42 deformations, work $A=A(x;y)$, ${D}_{f }$ = 2.86 and forces ${F}_{adh}={F}_{adh}(x;y)$, ${D}_{f}$ = 2.87 of adhesion) confirm the presence of size effects (2 < ${D}_{f}$<3). The average values of their main fractal parameters $\zeta$, $\eta$ и ${D}_{f}$ were obtained. This determines the relevance of conducting studies of size effects at the cellular level, since the use of the apparatus of fractal geometry makes it possible to predict the values of measured characteristics for statistically similar biological objects of larger or smaller sizes. In other words, the geometric and functional characteristics of cells of the same nature depend on their size. It is no secret that the linear dimensions of cells that are components of large biological systems (for example, human organs) usually vary over a very large range of measuring scales, for example, cells grow. As a result, rather large ensembles of statistically similar objects are formed, the values of both geometric and functional characteristics of which, depending on their size, change within a fairly wide range. Thus, the approaches and methods developed in this work make it possible to numerically find the geometric and functional parameters of not only similar objects of different sizes, but also to model the geometric and functional properties of the systems (organs) formed by them.

Mapping of the geometric and functional properties of the cell membrane surface also revealed the presence of a fairly large cellular structure with a cell size from 200 nm to 2 μm. More detailed studies of the cells, in turn, revealed their network structure from 50 to 350 nm, which most likely represents some “bags” of a disordered porous peptidoglycan gel—a three-dimensional network in a liquid medium. All this points to the complex helium-mosaic structure of the human buccal epithelium cell membrane, which largely determines its functional micromechanical characteristics.

For the first time, a non-trivial selective response of the membrane of a living adult cell of human buccal epithelium to an external micromechanical stimulus was studied at a high level of force effects ${F}_{const}$ and the presence of adhesive forces ${F}_{adh}$. The response of the membrane to the force *F*_*const*_ of an external local impact − the level of elastic (≤ 6 n*N*), active (6–21 n*N*), or passive (> 21 n*N*) stimulation is a non-trivial selective process and can exhibit a correspondingly elastic ($K=$ 67.4 N/m), active ($K$ = 80.2, the membrane reaction increases with load and is characterized by small plastic deformations) and passive ($K$ = 84.5 N/m, the reaction weakens with increasing load and is characterized by large plastic deformations) responses. In general, the values ${K=K(F}_{const})$ and ${E=E(F}_{const})$ depend on ${F}_{const}$. It was found that after overgoing slight plastic deformations ${\Delta h}_{stiff}$ < 300 nm, the membrane is able to independently restore its shape.

The fundamental disadvantages of AFM methods for studying cell membranes include the following: the need for preliminary preparation of samples for spatial fixation of individual cells on a substrate; the possible influence of the substrate on the properties of the cells located on it; high demands on the roughness of the substrate and its adhesive properties. The functional limitations of the method are mainly related only to the instrumental capabilities of the AFM and, in terms of spatial resolution, they are 10 nm in (*x;y*) and 2 Å in $z$, respectively, and in terms of micromechanical ones, Young's modulus is 1 kPa and, in terms of the elasticity coefficient, 0.056 N/m.

Nevertheless, the high accuracy and sensitivity of the method in a wide range of measuring scales while simultaneously measuring a wide range of functional characteristics, as well as the possibility of precise control of the degree of impact of the probe on the nanoobject under study at the micron and nanolevels, the availability of sufficiently advanced mathematical software makes this method unique and suitable for studying the relationship of geometric and functional characteristics in the local approximation at the micron and nanolevels also for other biological objects.

Thus, a fairly wide range of changes in the morphological and micromechanical characteristics of cell membranes of human buccal epithelium under normal conditions was revealed, depending on the level of external influence and response to an external micromechanical stimulus. Expansion of studies using AFM methods of functional and pathological changes in the morphology of buccal epithelial cells under the influence of external factors will significantly expand the capabilities and quality of this non-invasive diagnostic method and can become the basis for atomic and molecular nanoengineering (manipulation of nanoobjects, creation of micro- and nanostructured devices) both on the surface, as well as inside the cell.

## Data Availability

The datasets used and/or analyzed during the current study available from the corresponding author on reasonable request.
